# Americans do not select their doctors based on race

**DOI:** 10.3389/fsoc.2023.1191080

**Published:** 2024-01-24

**Authors:** Reilly Olinger, Benjamin Matejka, Rohan Chakravarty, Margaret Johnston, Eliana Ornelas, Julia Draves, Nishi Jain, Jane Hentschel, William Owen, Yuchuan Ma, William Marx, Joshua Freitag, Nicholas Zhang, Cameron Guage, Charles Crabtree

**Affiliations:** Dartmouth College, Hanover, NH, United States

**Keywords:** discrimination, doctors, conjoint experiment, experiment, bias, race, ethnicity

## Abstract

To what extent do Americans racially discriminate against doctors? While a large literature shows that racial biases pervade the American healthcare system, there has been no systematic examination of these biases in terms of who patients select for medical treatment. We examine this question in the context of the ongoing global COVID-19 pandemic, where a wealth of qualitative evidence suggests that discrimination against some historically marginalized communities, particularly Asians, has increased throughout the United States. Conducting a well-powered conjoint experiment with a national sample of 1,498 Americans, we find that respondents do not, on average, discriminate against Asian or doctors from other systematically minoritized groups. We also find no consistent evidence of treatment effect heterogeneity; Americans of all types appear not to care about the racial identity of their doctor, at least in our study. This finding has important implications for the potential limits of American prejudice.

## Introduction

To what extent do Americans racially discriminate against doctors? This question takes on particular importance during the ongoing COVID-19 pandemic, when more than 75.1 million people in America have been infected with the virus and more than 888,000 have died from it. With untold numbers of Americans likely to grapple with the long term, serious side effects of COVID-19 exposure (Del Rio et al., 2020), public demand for healthcare across the country is expected to remain high for years. Within this context, it is crucially important to understand how racial attitudes and discrimination might shape healthcare interactions and outcomes. In that vein, we examine how patients select medical care providers, when they have a choice, and what role, if any, racial biases play in their decisions.

An enormous, rich, and growing literature shows that racial attitudes influence many Americans’ decisions (Crabtree, 2018; Gaddis and Crabtree, 2021). We observe this robust stylized fact (Crabtree and Fariss, 2016) of racial discrimination in the USA in multiple contexts, such as in the labor market, where employers give individuals from historically minoritized groups fewer opportunities and pay them lower wages (Pager and Shepherd, 2008; Zschirnt, 2016; Quillian et al., 2017; Lippens et al., 2022, 2023; Quillian and Lee, 2023). We also observe it in housing, where landlords and bankers are more likely to pass them over for approval as renters and homeowners (Yinger, 1995; Flage, 2018; Gaddis et al., 2023), in credit decisions, where financial institutions reject them at higher rates and for them to pay higher interest rates, and in all manner of consumer interactions, where sellers quote them higher costs for goods and services (Ayres and Siegelman, 1995; Doleac and Stein, 2013). This discrimination also appears in politics, minoritized individuals receive less assistance and representation (Butler, 2014; Butler and Crabtree, 2017; Gell-Redman et al., 2018; Crabtree, 2019; Hughes et al., 2020; Block et al., 2021, 2022; Butler and Crabtree, 2021). Finally, and sometimes most profoundly, they experience this discrimination in the domain of law, where court systems and police levy against them greater penalties for legal infractions target them with repression even when they have done nothing wrong (Crabtree, 2019; Weaver and Prowse, 2020).

More relevant to our paper, an important line of work shows vast gaps in healthcare coverage, treatment, and outcomes across racial groups (Williams et al., 2019). Prior research, for example, has observed large differences in access to healthcare and in the health outcomes that result downstream (Higginson and Costantini, 2002; Kawachi et al., 2002; Graham, 2009; Cheon et al., 2016; Hibberd and Quan, 2017). Recent experimental work also indicates that doctors might have strong biases that can arise even in the context of critical lifesaving situations (Crabtree et al., 2022a). In line with this, studies have found that Black patients were significantly more likely to perceive racial discrimination in healthcare settings and that Asian immigrants were more likely than American-born Asian people and white people to perceive racial discrimination in the same setting (Lauderdale et al., 2006; Hausmann et al., 2011). Taken together, the findings from this literature suggest that race plays a key role in medical treatment, healthcare processes, and patient outcomes.

Despite a growing literature on the role that discrimination plays in healthcare and in public choices about goods and services, there has been relatively less research on *discrimination against healthcare workers*. This is a puzzling gap given the large body of research on racial inequalities in healthcare. While the focus of this literature has been on racial discrimination against patients, there are reasons to believe that discrimination also occurs against healthcare workers of all types, including doctors. Filut et al.’s (2020, p. 135) systematic literature review on biases in American healthcare finds that many physicians from systematically minoritized backgrounds believe that they have been discriminated against by patients. Their review covers 19 studies that involve discrimination by patients against healthcare providers. Most of these provide the results from interviews conducted with or focus groups conducted among doctors, such as Bhatt (2013) and Pololi et al. (2010). Others, like Coombs and King (2005) and Corbie-Smith et al. (1999), present the results of doctor surveys. The evidence from these studies suggests that doctors from minority groups in the United States, and elsewhere, often feel that they are discriminated against because of their background, harassed and passed up for promotions more than doctors from the majority group. While this literature has done much to raise the issue of racial discrimination against doctors in a variety of contexts, it is still developing and lacks concrete theoretical expectations about possible mechanisms that might be driving these biases.

Building on this mix of qualitative and quantitative accounts, and news reports about discrimination against Asian doctors during the COVID-19 pandemic (Bhanot et al., 2021), we provide the first empirical investigation of the degree to which racial biases influence individual choices about which doctor they would see for medical care. Our focus here is not on how individuals treat doctors of different characteristics once visiting them, but rather the extent to which the personal attributes of doctors shape whether individuals decide to see them at all. Based on prior findings about the pervasiveness of racial discrimination in American life, we theorize that when patients can select from several doctors, they might rely on their individual biases against groups in their decisions about who to seek for treatment.

We think that identifying these biases is especially important considering the damaging effect they can have on doctors (Leung et al., 2021), who must cope with the actions and decisions of their patients, and on discriminatory patients, who may opt for inferior care based on the doctor’s identity or race. If patients discriminate, this has concrete impacts on the job prospects, business successes, and even potentially the likelihood of dealing with malpractice suits for minority doctors. On the other hand, if patients do not discriminate at this point of medical interactions, this would suggest that American racial attitudes manifest at latter points in the healthcare process of potentially vary across contexts. The importance of our inquiry is further underscored by the fact that the American medical workforce continues to diversify—increasing the proportion of healthcare workers who might be discriminated against—and the likely reality that Americans post-COVID-19 will have greater medical needs - increasing the number of opportunities for individuals to engage in discrimination.

## Data and design

To test the extent to which Americans racially discriminate against doctors, we conducted a conjoint experiment with a national sample of 1,498 Americans recruited through Lucid Theorem with quotas for age, race, gender, educational attainment, household income, Census region, and political party. Lucid collected data from February 18 to March 4 2021. Conjoint experiments are commonly used in the social sciences to help understand how people value different attributes of possible choices (Hainmueller et al., 2015; Hainmueller and Hopkins, 2015; Auerbach and Thachil, 2018; Bansak et al., 2021; Jenke et al., 2021). Under a conjoint design, a researcher shows a survey or lab respondent a series of products—or a fictional doctor listing—and randomizes a set of potential product attributes—such as the attributes of the doctors in the listings. The objective of a conjoint experiment is to determine what combination(s) of a limited number of attributes is most influential in driving product choices; null effects are interpreted as respondents not using those attributes in their decision-making, while substantively and statistically significant effects suggest that attributes are important for respondents’ choices.

We chose to conduct a conjoint design about hypothetical doctors because this is, we think, the best available research design for studying the question at hand. This is true for at least two important reasons. First, the main alternative to a conjoint experiment—looking at the actual patterns in doctor visitation across various demographic groups—would have potential limitations. With this research design, we could not hold all other factors about the doctor constant. If we observed differential rates of doctor visitations across different doctor attributes, we could not know if these were caused by these attributes or other unobservable features. Ignoring this fundamental problem to making inferences about racial discrimination against doctors using observational data, there is another larger concern: no comprehensive dataset like this exists. Based on these two reasons alone, we think that an experiment about fictional doctors is the most appropriate approach to examine racial discrimination in doctor selection. In addition, though, we think that conjoint experiments are a particularly powerful tool for understanding racial discrimination against doctors. This is because the experimental literature shows that conjoint experiments can be used to minimize satisficing (i.e., when respondents complete surveys as quickly as possible), demand effects (i.e., when respondents answer as they think researchers want them to), and social desirability bias (i.e., when respondents provide socially acceptable responses instead of their true responses) (Horiuchi et al., 2022).

We implement our survey with members of the public because they are likely patients. In addition to understanding public preferences as patients, it is also important to understand if members of the public exhibit racial discrimination to doctors since both the behavior of elected officials (Healy and Malhotra, 2013) and the behavior of healthcare providers may be shaped by the preferences (Morgan and Campbell, 2011; Baicker et al., 2014; Clinton and Sances, 2018). This means that any biases exhibited by the public might have diffuse consequences when it comes to public health priorities and even potentially medical care staffing and practices.

In our conjoint experiment, we ask respondents to choose between one of two possible doctors for medical care. It is reasonable to ask our respondents to evaluate doctors this way because many of them will likely need medical care in the next year, which contributes to the ecological validity of our experiment. We randomized several characteristics of the doctors based on our understanding of what factors might play into patient decisionmaking.

First, we randomized the reported Yelp review score (2.8/5, 3.9/5, 5/5) for each doctor. We selected these values by scraping Yelp for all reviews of doctor ratings and then picking the 25, 50, and 75% quantiles. We include this because of the central role that review ratings play in American selections of goods service providers, and because it allows us to test whether respondents are attentive to the doctor profiles and are responding in a way that is consistent with what we already know about public preferences. If our experiment were ecologically valid (i.e., if our experiment represented a process that people complete in the “real” world), we would expect that the effect of review ratings would be both statistically significant and substantively large. Another reason that we chose to manipulate this factor is that it allowed us to account for a potentially important form of bias in our experimental design. Specifically stating the doctors’ rating allowed us to account for possible differential biases in the perceptions of ratings based on other individual characteristics. By providing this information to possible patients directly, we are potentially attenuating a mechanism that might drive discrimination. Since our (necessary) manipulation check blocks a potential channel for discrimination (i.e., one that arises from misperceptions about the overall quality that differ across race and other attributes), it allows us to estimate a vitally important quantity of interest—that is, the extent to which individuals exhibit racial discrimination independent from this form of statistical discrimination (Schwab, 1986; Guryan and Charles, 2013). We acknowledge though that our findings here are limited to the type of reviews we used (i.e., Yelp) and that other review sources might have led to different reactions.

In addition to the Yelp review rating for each doctor, we also randomized the doctor’s age (35, 41, 47, 60, 66), gender (man, woman), medical-degree granting institution (Drexel, East Carolina, Harvard, Michigan State, Tufts, UCLA), and race (we term these conditions White, Hispanic, Black, or Asian). We also randomized the type of clinic that the doctor practiced at (small public, large public, small private, large public) and the expected wait time (10 min, 15 min, 20 min). We selected these attributes based on prior literature about doctor selection. We directly stated each doctor’s age, educational background, type of clinic in which they worked, and expected wait time. In line with the common practice in audit studies (Butler, 2014; Costa, 2017; Gaddis, 2017), we manipulated gender and race through the names we gave our fictional doctors (Crabtree, 2018; Crabtree and Chykina, 2018; Crabtree et al., 2022b). To help ensure that survey respondents received the correct racial manipulation, we build on the largest known survey of American perceptions about names to date (Crabtree et al., 2022c), selecting only those names that survey respondents (a) correctly perceived the intended race of at least 90% of the time, (b) thought had a college degree or higher, and (c) thought belonged to an American citizen at least 90% of the time.

We randomized this set of attributes because these are either often considered to be important when patients select doctors (e.g., review ratings, wait time, clinic type, age, and education see Salisbury, 1989; Santos et al., 2017), have been shown to inform decision making in other social contexts (e.g., gender and race; see Ayres and Siegelman, 1995; Paxton et al., 2007; Kalkan et al., 2009; Butler, 2014; Costa, 2017), and/or have well-documented inequities in the health domain (see Higginson and Costantini, 2002; Kawachi et al., 2002; Graham, 2009; Cheon et al., 2016; Hibberd and Quan, 2017). All of the attributes in our conjoint designs are information that individuals could know about their doctors with some consumer research.

The COVID-19 pandemic is an important context in which to conduct this experiment. The spread of COVID-19 has increased the salience of medical care and treatment. Most Americans must consider this possibility of seeing a doctor daily. Another reason why it is an important context relates to patterns of racial discrimination in the United States since the early days of the pandemic, when a wealth of evidence showing that discrimination against some systematically minoritized groups has increased substantially.

Finally, we note that in our analyses, we focus on main treatment effects (i.e., among our entire sample) and on conditional effects based on the self-reported political party of the individuals in our study and their race (to be thorough, however, we examine treatment effect heterogeneity by all other available baseline characteristics in the Supplementary material).

## Results

**Figure 1:**
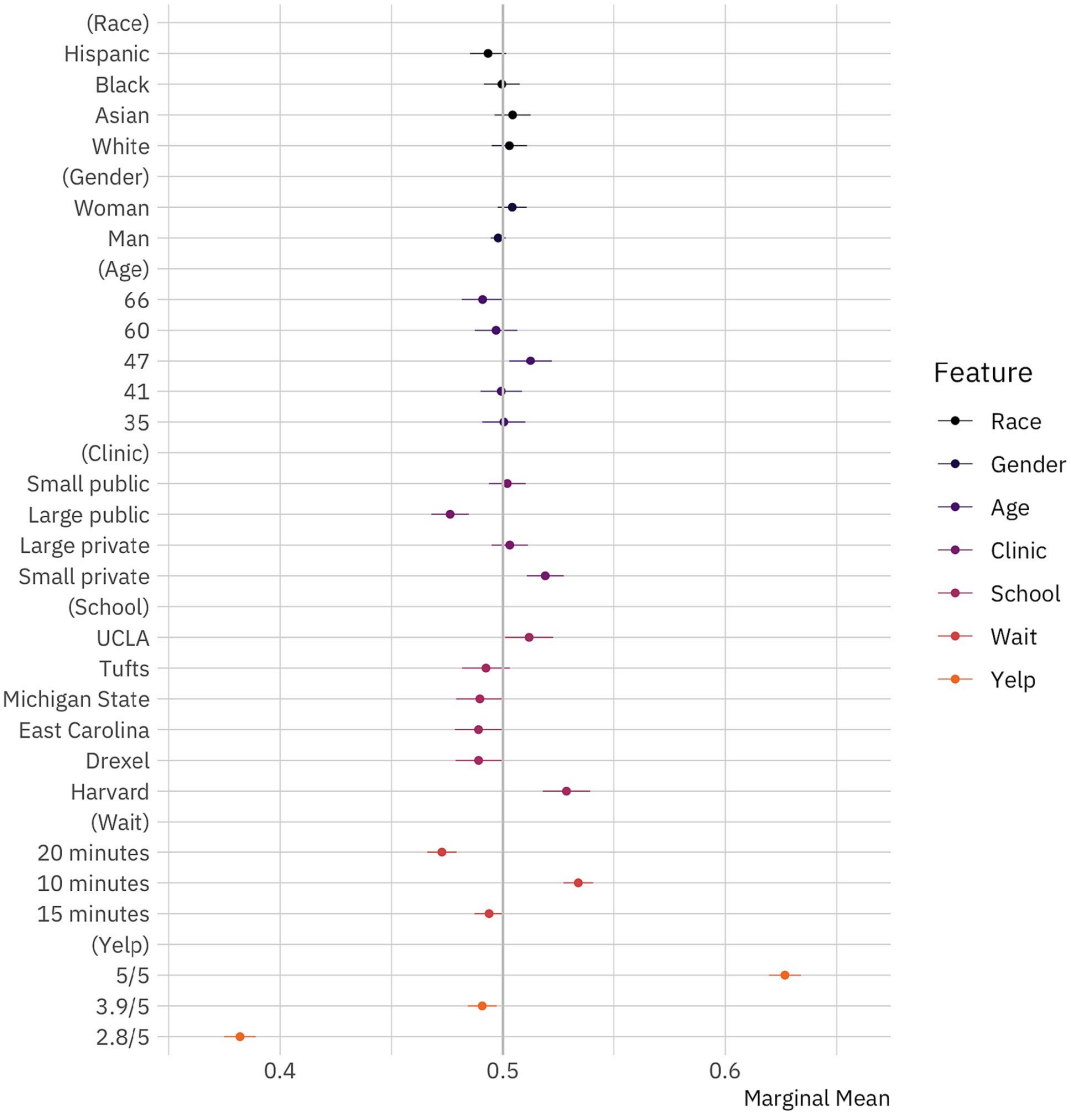
Public preferences for doctors. Marginal means plot for the effect of doctor attributes on survey respondent selection. The circles represent the marginal means while the thin bars denote 95% confidence intervals. Coefficients on the left side of the gray line at 50% indicate that respondents are, all-else-equal, less likely to choose a doctor with the given characteristics on the vertical axis; those on the right are, all-else-equal, more likely to choose a doctor with the given characteristic. The unit of analysis is the respondent-choice profile. Hence, the N reported in our models below is the number of respondents (1,498) multiplied by the number of pairwise choices (15) and individuals within those pairs (2). *N* = 44,940. Confidence intervals are calculated based on standard errors clustered by respondent (Bansak et al., 2021).

Figure 1 shows the results from our conjoint experiment; circles denote marginal means and thin bars denote 95% confidence intervals. Confidence intervals are calculated based on standard errors clustered by respondent (Bansak et al., 2021), since we have multiple observations per respondent. Conditional marginal means are grouped by experimental factor and denote the predicted probability for a doctor with a specific factor level, across all other factor levels. If the confidence intervals for a doctor attribute cross the 50% line (the gray vertical reference line), we cannot reject the null hypothesis that patient choices in regards to that factor level are random. In other words, we cannot be sure that patients discriminate either *against* or *for* doctors with that characteristic. Importantly, if individuals were selecting doctors at random, we would not expect them to exhibit any biases against or toward certain.

We first examine whether survey respondents were more likely to select doctors with higher Yelp ratings, since this provides some insight into the way individuals interacted with our conjoint experiment. If respondents were paying attention to it and taking it seriously, we would expect them to select doctors with better reviews. Specifically, we would expect that the marginal means would increase with ratings and that the differences would be substantially large. In fact, this is just what we see. Survey respondents indicate that they would see doctors with 5/5 ratings about 63% [61.96–63.39%, 95% CI] of the time, doctors with 3.9/5 ratings about 49% [48.42–49.71%] of the time, and doctors with 2.8/5 ratings about 38% [37.49–38.92%] of the time.

We next turn to whether survey respondents select doctors based on other characteristics. If our respondents were making decisions based solely on a doctor’s rating, we would expect that all other characteristics manipulated in our conjoint experiment—age, clinic type, educational background, gender, and wait time—would be insignificant. Particularly, if respondents were treating individuals from all racial groups the same—responding to our racial conditions randomly—we would expect to see no differences across this dimension.

We find that some of the characteristics matter while others do not. Specifically, we find evidence that respondents are sensitive to the potential wait times that they would face, preferring 10 min (53% [52.71–54.04%]) or 20 min (47% [46.60–47.91%]). They are indifferent between doctors when the wait time is 15 min (49% [48.72–50.04%]). These findings add additional credibility to our experimental design, as they indicate that respondents are reacting to the choices they face as we would generally expect them to in the real world. We also observe that educational background seems to matter. While a medical degree from Michigan State (49% [47.89–50.03%]) or Tufts (49% [48.17–50.31%]) does not decrease the likelihood that respondents would pick a doctor, a degree from Drexel (49% [47.87–49.94%]) or East Carolina (49% [47.84–49.97%]) does, though to a very small extent. On the other hand, degrees from Harvard (52% [51.78–53.92%]) and UCLA (51% [50.09–52.25%]) bolster a doctor’s chances of being chosen, though not by much. Taken together, these educational results also fit with what we should expect about respondent behavior. Looking at the results for clinic type, we see that respondents prefer doctors who practice in small private clinics (52% [51.07–52.72%]), would rather avoid those who practice at large public clinics (48% [46.78–48.47%]), and are indifferent to small public and large private clinics. These findings fit broadly with past work on patient preferences.

Examining the effects of personal characteristics, we see that age appears to matter to some extent. Individuals appear not to care about how old doctors are most of the time, with the one exception being a weak preference for 47-year-olds (51% [50.28–52.19%]). We interpret this to indicate that individuals prefer doctors who have some mix of youth and experience. More interestingly, we find that respondents do not prefer doctors of one gender to another. The marginal means for both men and women doctors are 49.8% [49.44–50.12%] and 50.4% [49.76–51.07%], respectively, and both estimates are statistically indistinguishable from 50% (*p* > 0.2). In other words, respondents do not seem to consider the gender of doctors, holding all else constant, when making healthcare decisions. While women face discrimination in many aspects of American life (Davis and Greenstein, 2009), and face discrimination by gatekeepers within the medical profession (Kouta and Kaite, 2011), it seems that prospective patients are willing to set aside their gender attitudes when selecting doctors to provide care.

Finally, turning to the effects of race, our central interest in this paper, we find that respondents *do not appear* to discriminate against systematically minoritized groups. While the marginal mean for Hispanics is slightly lower than it would be by chance (49.33% [48.52–50.14%]), we cannot reject that this effect differs from 50% (*p* > 0.1). Comparing this effect to results from audit studies (Costa, 2017; Quillian et al., 2017; Gaddis et al., 2023) or previous conjoint experiments (Hainmueller et al., 2015; Hainmueller and Hopkins, 2015; Auerbach and Thachil, 2018; Bansak et al., 2018; Jenke et al., 2021), we also see that this effect is very small. At the least, we can say that any racial discrimination that Hispanic doctors face in this particular context appears tiny in comparison to the racial discrimination faced by them and other systematically minoritized in America across different contexts (Gaddis et al., 2023). Importantly, we also find--albeit surprisingly, given the increasing evidence of anti-Asian discrimination in America (Golder 2023) that respondents do not appear to discriminate against Asian (50.43% [49.62–51.24%], *p* > 0.29) or Black (50% [49.14–50.76%], *p* > 0.9) doctors. Conversely, respondents do not appear to prefer White doctors (50.28% [49.49–51.08%], *p* > 0.47). We find no evidence of racial or gender discrimination in doctor choice. For more information see Supplementary material. Taken together, our results suggest that racial biases do not influence doctor selection in America when respondents are presented with a wealth of information about their options. This potentially offers insight into the limits of American prejudice.

**Figure 2:**
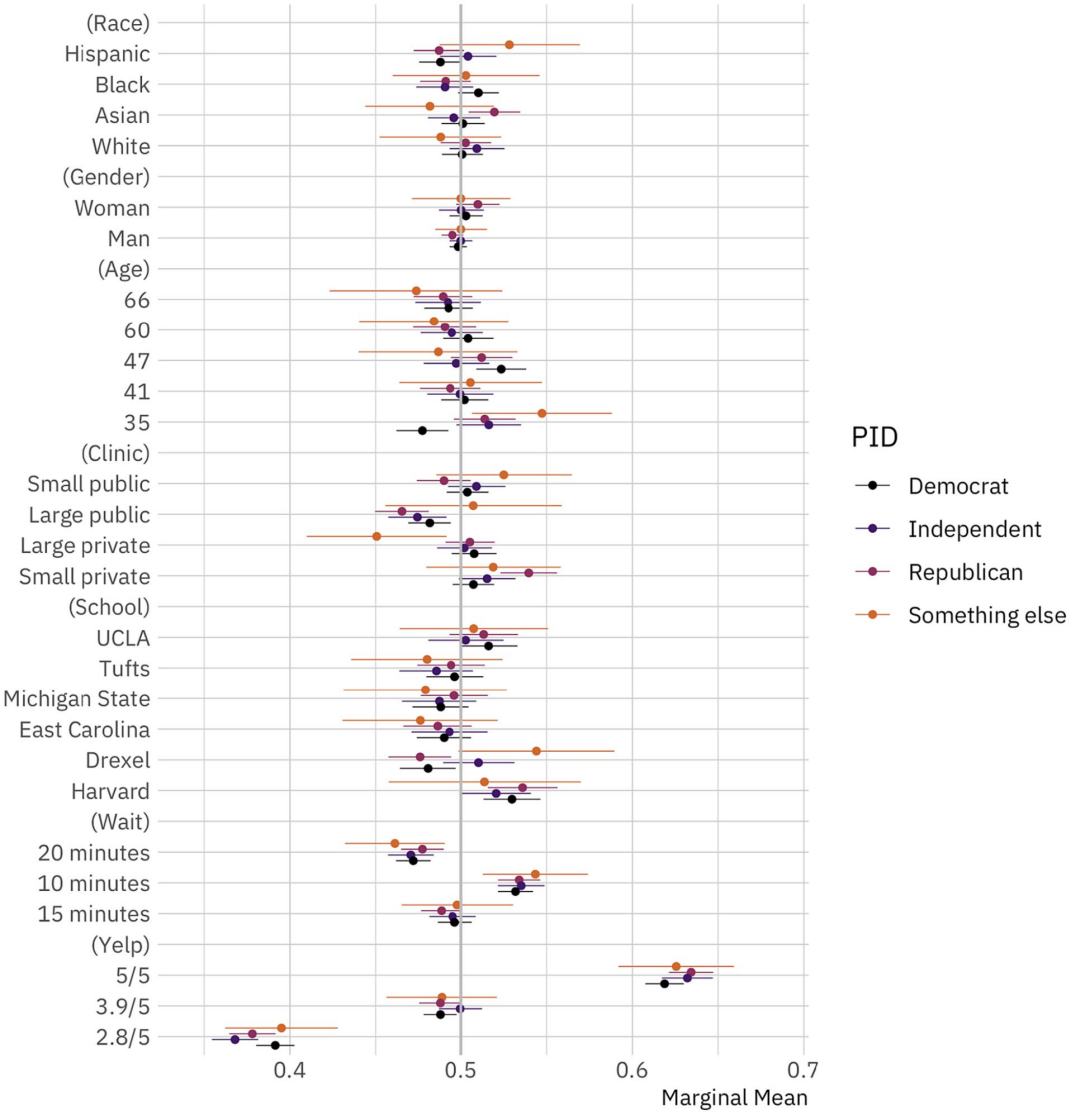
Public preferences for doctors by political identification. Conditional marginal means for the effect of doctor attributes on survey respondent selection by political identification. The circles represent the marginal means while the thin bars denote 95% confidence intervals. Coefficients on the left side of the gray line at 50% indicate that respondents are, all-else-equal, less likely to choose a doctor with the given characteristics on the vertical axis; those on the right are, all-else-equal, more likely to choose a doctor with the given characteristic. The unit of analysis is the respondent-choice profile. The N reported in our models below is the number of respondents (1,498) multiplied by the number of pairwise choices (15) and individuals within those pairs (2). *N* = 44,940. Confidence intervals are calculated based on standard errors clustered by respondent (Bansak et al., 2021).

Perhaps these results mask substantial heterogeneity across respondents in our sample. Put differently, individuals from some groups might exhibit more or less racial discrimination. Here we examine the extent to which respondent political leanings influence how they respond to our conjoint experiment, in general, and how they react to doctors of different races, specifically. Figure 2 shows the results of this model. As in Figure 1, circles denote marginal means and thin bars denote 95% confidence intervals. Plotted points and confidence intervals differ in color based on political identification. Marginal means are grouped by experimental factor. As a reminder, if the confidence intervals for a doctor attribute cross the 50% line (the gray vertical reference line), we cannot reject the null hypothesis that patient choices in regards to that attribute are random.

We would expect that public preferences about doctors should vary based on political identification. Contemporary American society is marked by high levels of political polarization (Fiorina and Abrams, 2008; Iyengar et al., 2019). In line with that, prior work has shown that political identification might shape everything from where Americans live (Bishop, 2009; Shafranek, 2021 though see also Gimpel and Hui, 2015; Mummolo and Nall, 2017), to who they choose to date and marry (Nicholson et al., 2016; Huber and Malhotra, 2017; Hersh and Ghitza, 2018), how they conduct economic transactions (Neilson, 2010; Engelhardt and Utych, 2020; Kam and Deichert, 2020), and how they perceive racial, ethnic, and religious minorities (Giles and Hertz, 1994; Barreto and Bozonelos, 2009; Kalkan et al., 2009; Enos, 2016; Haner et al., 2020; Lajevardi, 2020).

What we find, though, is the opposite. It seems that political leanings shape what Americans perceive and what they do in many areas of life but *not* what they look for in their doctors. There is weak evidence that Republicans and Democrats prefer not to visit Hispanic doctors, but these findings are relatively small and statistically insignificant (*p* > 0.10). There is also some evidence that Republicans prefer Asian doctors. But, overall, the differences across political identification are not statistically significant or substantively important. This is a striking finding given the large, growing literature reference above that shows the central importance of political identification in driving American perceptions and choices.

**Figure 3:**
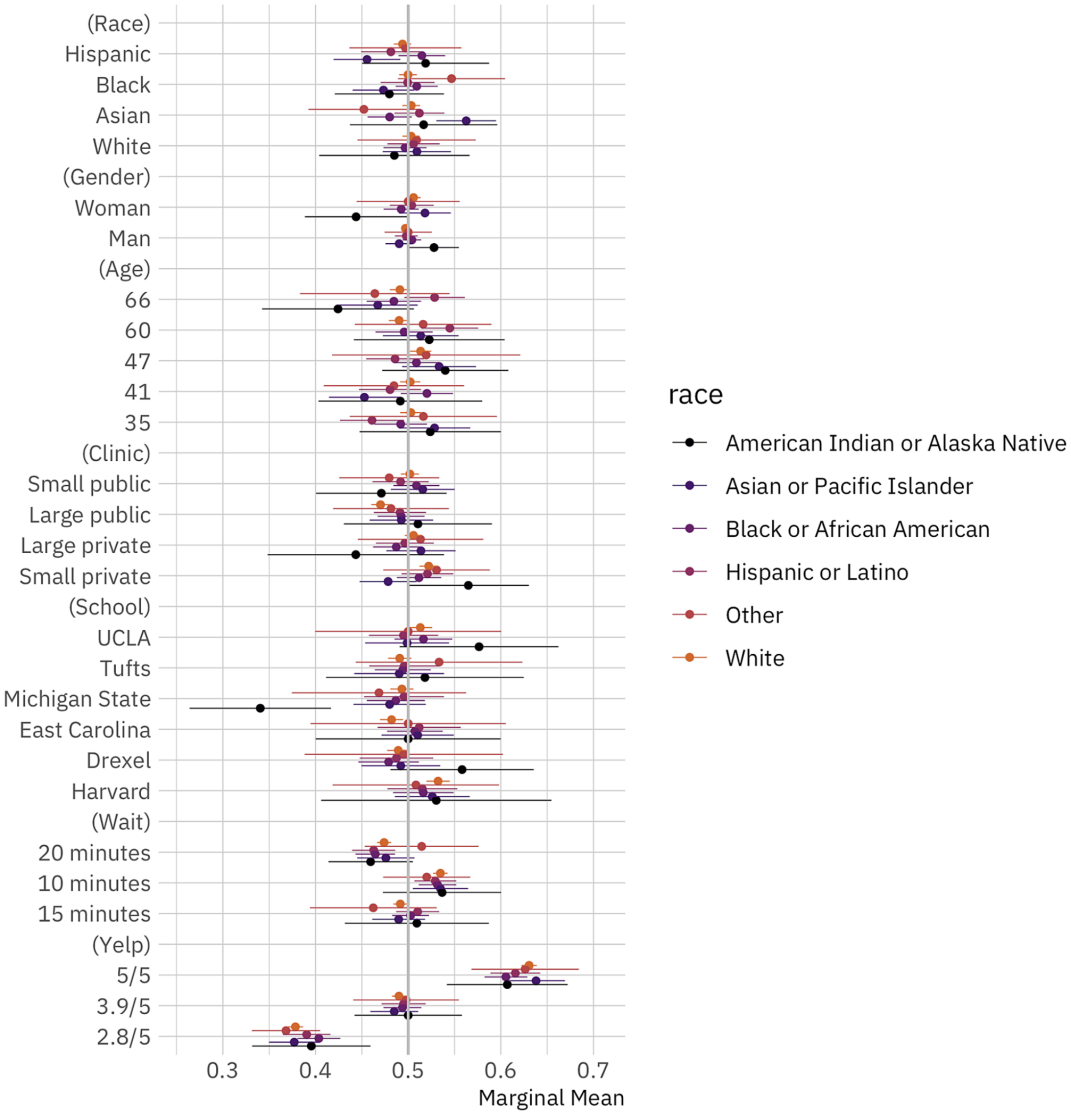
Public preferences for doctors by race. Conditional marginal means for the effect of doctor attributes on survey respondent selection by respondent race. The circles represent the marginal means while the thin bars denote 95% confidence intervals. Coefficients on the left side of the gray line at 50% indicate that respondents are, all-else-equal, less likely to choose a doctor with the given characteristics on the vertical axis; those on the right are, all-else-equal, more likely to choose a doctor with the given characteristic. The unit of analysis is the respondent-choice profile. The N reported in our models below is the number of respondents (1,498) multiplied by the number of pairwise choices (15) and individuals within those pairs (2). *N* = 44,940. Confidence intervals are calculated based on standard errors clustered by respondent (Bansak et al., 2021).

One might reasonably expect, though, that race is a more salient group characteristic than political leanings in this context. In line with that, we also provide the marginal means for our conjoint attribute levels by respondent race in Figure 3. As in Figure 2, circles denote marginal means and thin bars denote 95% confidence intervals. Plotted points and confidence intervals differ in color based on political identification. Marginal means are grouped by experimental factor. Again, as a reminder, if the confidence intervals for a doctor attribute cross the 50% line (the gray vertical reference line), we cannot reject the null hypothesis that patient choices in regards to that attribute are random.

There are several interesting empirical findings across respondent racial groups. While Black and white respondents exhibit no meaningful preferences for doctors depending on their race (i.e., differences between marginal means for race attribute levels are not statistically significant), Asian and Hispanic respondents do exhibit some striking, and similar, racial preferences. Asian respondents are more likely to select Asian doctors and less likely to select Black doctors, while Hispanic respondents are more likely to select Black doctors and less likely to select Asian doctors. In other words, while we find that respondents on average have no racial preferences in doctor selection, and that Black and white respondents have no racial preferences either, we do find that Asian and Hispanic respondents seem to take race into account when selecting possible providers.

In the Supplementary material, we further explore possible treatment effect heterogeneity by looking at group differences across respondent education, gender, and views on racial discrimination. We find no compelling, consistent differences across groups, especially after adjusting *p*-values for multiple comparisons. Ultimately, while there is some small variation in our treatment effect heterogeneity results, the overall trend is clear: members of the public do not generally provide differential treatment to doctors in our task based on race.

## Discussion

Americans regularly interact with doctors, yet we do not know as much as we might about what racial biases, if any, drive these encounters and the extent to which they might influence practitioner choice. Our study, based on a conjoint experiment conducted with a national quota-based sample of 1,498 Americans, provides new evidence that patients in the United States do not appear to engage in racial discrimination when (and we emphasize this conditional statement) choosing doctors for a potential visit. The results of our experiment stand out against the ever-growing number of studies showing that racial discrimination marks most aspects of daily life in America.

We think that there are at least five reasons why we do not observe racial discrimination, in general, or anti-Asian discrimination, within the particular context of our study. First, and we think most significantly, our null results for racial discrimination are likely null results for taste-based discrimination. It could be that patients often engage in statistical discrimination when evaluating doctors—for instance, making assumptions about things like where doctors went to school based on their race—and that this drives some of the discrimination that’s been observed in other studies. By providing this information in our conjoint experiment, we are potentially eliminating one important mechanism (i.e., statistical discrimination), that could lead to discrimination. That said, we should note that while our conjoint design provides information about several attributes of our fictional doctors, attributes that patients typically consider important while evaluating care providers, it does not provide all possible information about the doctors. This means that statistical discrimination might still occur through other omitted attributes. On the whole, though, we think that our research design allows us to partially disentangle statistical and taste-based discrimination, and that our results provide suggestive evidence that the public does not appear to have a taste for race-based discrimination in doctor selection.

Second, we only focus on doctor choice. It is possible that doctors from systematically minoritized racial backgrounds are more likely to be discriminated against and even abused during clinical encounters by the very same patients who chose them, or that they may be more likely to receive complaints or a lawsuit. The small but growing literature about racism against health professionals would support this.

Third, the public views doctors differently than other individuals. According to a Pew study conducted in January 2019, 74% of the American public have a mostly positive view of doctors, while another 68% expressed a mostly positive view of medical research scientists. The positive approval rating of doctors has only likely increased since the COVID-19 pandemic began, especially in light of the positive coverage they have received in news media. Perhaps the positive perceptions that Americans have of doctors overshadow any negative perceptions that they might have of systematically minoritized racial groups.

Fourth, it might be that individuals recognize - at a subconscious level at least - that engaging in racial discrimination entails costs and leads to less efficient outcomes. Potentially people are willing to pay these costs in some aspects of their lives, but not when their health might be jeopardized. This would suggest that racial biases and discrimination might be more common in low-cost interactions. We think that this is a promising avenue for future research.

Fifth, we might not find anti-Asian discrimination, in particular, be due to widespread views of Asians as a “model minority” (Chou and Feagin, 2015). This problematic view treats Asian Americans as a homogenous, successful group, glossing over achievement disparities and labor market disadvantages within the racial category (Sakamoto et al., 2012). This widespread and frequently perpetuated stereotype could have lead respondents to have potentially elevated expectations for the care that Asian American doctors could provide and, therefore, a positive bias toward them. In other words, they might have associated Asian doctors with having higher levels of ability, even holding constant educational background.

We acknowledge several limitations of our work and think that they should animate additional research on the contexts in which Americans discriminate against systematically minoritized racial groups.

First, there is an open question about the degree to which we might expect respondent choices in surveys to map on to real-world behavior. The evidence for whether hypothetical choices match actual behavior is mixed, but there’s also evidence that this link is stronger with conjoint experiments than other types of survey question items or experimental designs, as we discuss above (Bansak et al., 2021). We think that future work might want to consider ways of increasing the external validity of our design, potentially by fielding some sort of field experiment to answer this research question.

Second, we conducted our survey experiment in March 2021, in the middle of an ongoing global pandemic. As we discussed above, this temporal context might have caused respondents to view doctors of all races in an equally positive hue. Second, we focus here on how potential patients might choose doctors in the absence of pressing medical needs. It might be that when patients are actually experiencing physical discomfort or pain, or stress-related to those conditions, they are less likely to evaluate medical options objectively and more inclined to rely on deep-seated biases.

Third, while we used age, ethnicity, income, education, and race quotas to ensure a nationally representative sample at the respondent recruitment stage, we introduced some demographic skew into our sample when we dropped inattentive subjects. As a result, our sample had too many white people (+15.28% over the national population) and higher-income respondents (+2.77%), and too few Black people (−3.105%), LatinX people (−11.877%), and lower-income respondents (−3.079%).

Fourth, it is well understood that the findings from conjoint experiments are specific to the attributes randomized. This means that our results might have changed if we would have included a different set of factors other than doctor race. We think that one reason why we might not have found evidence for bias here is because we provided explicit information about doctor quality. Absent that, respondents might have used racial signals to draw discriminatory inferences about where doctors graduated from and the quality of their practice. In sum, researchers should probe the temporal validity of our work in subsequent studies, assess the extent to which individuals experiencing medical issues might be more likely to exhibit bias, and check the generalizability of our findings to other samples and conjoint designs.

Fifth, our data and results are specific to national sample of Americans, and we do now know the extent to which they might generalize to other countries. This is a difficult question to answer, as racial and ethnic cleavages differ across nations, both in the groups involved but also in the sharpness of the divisions. We can only speculate, as our data do not travel beyond American borders, but we might think that our results could generalize to other countries if patients prioritize care over their own biases.

More broadly, scholars concerned about racial discrimination should continue investigating its presence and drivers in healthcare. Given the ongoing pandemic, and the societal aging trends in many developed countries, we can expect public interactions with medical personnel to increase greatly in the years and decades ahead and, consequently, the number of chances for discrimination to increase correspondingly. In attempting to understand how racial discrimination influences medical choices and outcomes, we can shed light on group dynamics in a vital, growing context.

## Methods

### Data and participants

In determining whether Americans discriminate against doctors based on their race or ethnicity, three studies were conducted: two pilot studies (*n* = 174, *n* = 330) and a final study (*n* = 1,498). All surveys were programmed in Qualtrics. Participants were recruited through Prolific for the pilot studies and through Lucid Theorem for the final study. Quotas on age, gender, race/ethnicity, party identification, and education were used in all three sample recruitments.

### Survey instrument

After confirming respondents’ consent to participate in our study, we conducted a two-step attention check (Aronow et al., 2020). Following this attention check, we collected socio-demographic data about the respondents’ political affiliation and ideology, gender identity, country of birth, American citizenship status, race/ethnicity, annual household income and education level. We then asked respondents whether they agreed that racial discrimination is a major problem in the United States.

Following these background questions, participants completed a vignette experiment where they were assigned to one of three treatments. In one, they read nothing. In the second, they read about a fictional incident of racial violence against Asian Americans that was presented as a real news excerpt. It centered on the story of an Asian American family who had their family restaurant attacked by racists. In the third, they read an automatically generated placebo condition (Porter and Velez, 2022). This treatment was created using Open AI’s novel large-scale unsupervised language model GPT-2 based on the seed phrase, “Jane Smith and her brother, Joe, showed up at their family’s restaurant.” Following Porter and Velez’s recommendations, we generated a large number of these vignettes (*n* = 4,930) and randomly assigned one of them to recipients who received our placebo condition. After reading the placebo and fictional news vignettes, respondents were asked to write a bit about how that made them feel. The idea here was that those who read the excerpt about racial violence against Asian Americans would be forced to engage in perspective taking and thus exhibit less discrimination against Asian doctors. We find and show in the Supplementary material that none of these treatments influenced responses, probably because (as we explained above) we observe no discrimination against Asian doctors, so we do not focus on that part of the design or results in this paper.

After completing the vignette experiment, participants completed a conjoint experiment focusing on doctor choice. We asked them to evaluate two possible doctors and choose between them based on which they would be more likely to visit. We had them complete this task 15 times. We presented participants with information about the doctors in a table, varying six attributes. As described above, we presented information on (1) the doctor’s name, (2) their age, (3) the medical school they attended, (4) the clinic type where the doctor practices, (5) the average wait time and (6) the doctor’s rating on a five-star basis. The order of attributes in the table was fixed, but the levels of attributes were randomized.

We selected names to signal racial identities based on Crabtree et al. (2022b). Our age levels were based on 2018 age distribution data of U.S.-based physicians. We randomly selected medical schools across the 2020 U.S. News World and Report ranking - UCLA, Tufts, Michigan State, East Carolina, Drexel, Harvard. Clinic type was limited to “small public,” “small private,” “large public,” and “large private,” capturing broad differences in medical practice time. Physician wait time was limited to 10, 15 and 20 min, a range of reasonably plausible values.

To construct our Yelp ratings levels, we scraped all results for the query “Doctors” in American cities with populations larger than 100,000 (*N* = 382) according to the US Census. Using this procedure, we constructed a sample size of 109,551 practices; 61,021 contained a rating. We calculated averages and standard deviations for physician practices at the city level, then averaged to the national level, weighted by the cities’ populations. We then calculated the 25, 50, and 75% quantiles of the resulting distribution and used those values in our conjoint.

### Analytic strategy

To determine the effect of each experimental factor in the conjoint, we calculated marginal means for the whole sample and for subgroups. Confidence intervals are calculated based on standard errors clustered by respondent (Bansak et al., 2021).

### Robustness checks

We find similar results when we calculate average marginal component effects (AMCEs) instead of marginal means. Our results are also surprisingly robust across subgroups, as mentioned above. In addition, we obtain similar findings if we include respondents who failed the attention check and if we drop those who used a mobile device to complete our survey.

## Data availability statement

The datasets presented in this study can be found in online repositories. The names of the repository/repositories and accession number(s) can be found here: https://dataverse.harvard.edu/dataset.xhtml?persistentId=doi:10.7910/DVN/KFQYTJ.

## Ethics statement

The studies involving humans were approved by Dartmouth College Committee for the Protection of Human Subjects. The studies were conducted in accordance with the local legislation and institutional requirements. The participants provided their written informed consent to participate in this study.

## Author contributions

RO, BM, RC, MJ, EO, JD, NJ, JH, WO, YM, WM, JF, NZ, CG, and CC: conceptualization, methodology, investigation, and writing—original draft. WM and CC: visualization and writing—review & editing. CC: supervision. All authors contributed to the article and approved the submitted version.

## References

[ref1] Aronow P. M. Kalla J. Orr L. Ternovski J. (2020). Evidence of rising rates of inattentiveness on Lucid in 2020.

[ref2] Auerbach A. M. Thachil T. (2018). How clients select brokers: Competition and choice in India's slums. Am. Polit. Sci. Rev. 112, 775–791. doi: 10.1017/S000305541800028X

[ref3] Ayres I. Siegelman P. (1995). Race and gender discrimination in bargaining for a new car. Am. Econ. Rev. 85, 304–321.

[ref4] Baicker K. Finkelstein A. Song J. Taubman S. (2014). The impact of Medicaid on labor market activity and program participation: evidence from the Oregon Health Insurance Experiment. Am. Econ. Rev. 104, 322–328. doi: 10.1257/aer.104.5.322, PMID: 25177042 PMC4145849

[ref5] Bansak K. Ferwerda J. Hainmueller J. Dillon A. Hangartner D. Lawrence D.. (2018). Improving refugee integration through data-driven algorithmic assignment. Science 359, 325–329. doi: 10.1126/science.aao4408, PMID: 29348237

[ref6] Bansak K. Hainmueller J. Hopkins D. J. Yamamoto T. (2021). “Conjoint survey experiments” in Advances in Experimental Political Science, vol. 19, 19–41.

[ref7] Barreto M. A. Bozonelos D. N. (2009). Democrat, Republican, or none of the above? The role of religiosity in Muslim American party identification. Polit. Religion 2, 200–229. doi: 10.1017/S1755048309000200, PMID: 38034472

[ref8] Bhanot D. Singh T. Verma S. K. Sharad S. (2021). Stigma and discrimination during COVID-19 pandemic. Front. Public Health 8:577018. doi: 10.3389/fpubh.2020.577018, PMID: 33585379 PMC7874150

[ref9] Bhatt W. (2013). The little brown woman: Gender discrimination in American medicine. Gen. Soc. 27, 659–680. doi: 10.1177/0891243213491140, PMID: 25416685

[ref10] Bishop B. (2009). The big sort: Why the clustering of like-minded America is tearing us apart. New York, NY: Houghton Mifflin Harcourt.

[ref11] Block R. Jr. Crabtree C. Holbein J. B. Monson J. Q. (2021). Are Americans less likely to reply to emails from Black people relative to White people? Proc. Natl. Acad. Sci. 118:e2110347118. doi: 10.1073/pnas.2110347118, PMID: 34930841 PMC8719855

[ref12] Block R. Jr. Crabtree C. Holbein J. B. Monson J. Q. (2022). Reply to Mitterer: Conceptual and empirical issues that arise when using correspondence audits to measure racial discrimination. Proc. Natl. Acad. Sci. 119:e2210695119. doi: 10.1073/pnas.2210695119, PMID: 35972976 PMC9499501

[ref13] Butler D. M. (2014). Representing the advantaged: How politicians reinforce inequality. Cambridge, MA: Cambridge University Press.

[ref14] Butler D. M. Crabtree C. (2017). Moving beyond measurement: Adapting audit studies to test bias-reducing interventions. J. Exp. Polit. Sci. 4, 57–67. doi: 10.1017/XPS.2017.11

[ref15] Butler D. M. Crabtree C. (2021). “Audit studies in political science” in Advances in Experimental Political Science, vol. 42, 42–55.

[ref16] Cheon S. Agarwal A. Popovic M. Milakovic M. Lam M. Fu W.. (2016). The accuracy of clinicians’ predictions of survival in advanced cancer: a review. Ann. Palliat. Med. 5, 22–29. doi: 10.3978/j.issn.2224-5820.2015.08.04, PMID: 26841812

[ref17] Chou R. S. Feagin J. R. (2015). Myth of the model minority: Asian Americans facing racism. New York, NY: Routledge.

[ref18] Clinton J. D. Sances M. W. (2018). The politics of policy: The initial mass political effects of medicaid expansion in the states. Am. Polit. Sci. Rev. 112, 167–185. doi: 10.1017/S0003055417000430

[ref19] Coombs A. A. T. King R. K. (2005). Workplace discrimination: experiences of practicing physicians. J. Natl. Med. Assoc. 97:467. Available at: https://www.ncbi.nlm.nih.gov/pmc/articles/PMC2568696/ PMID: 15868767 PMC2568696

[ref20] Corbie-Smith G. Frank E. Nickens H. W. Elon L. (1999). Prevalences and correlates of ethnic harassment in the US Women Physicians’ Health Study. Acad. Med. 74, 695–701. doi: 10.1097/00001888-199906000-00018, PMID: 10386100

[ref21] Costa M. (2017). How responsive are political elites? A meta-analysis of experiments on public officials. J. Exp. Polit. Sci. 4, 241–254. doi: 10.1017/XPS.2017.14

[ref001] Crabtree C. Fariss C. J. (2016). Stylized facts and experimentation. Sociological Science. 3, 910–914.

[ref22] Crabtree C. (2018). “An introduction to conducting email audit studies” in Audit studies: Behind the scenes with theory, method, and nuance, 103–117.

[ref23] Crabtree C. (2019). Measuring and explaining discrimination. Diss.

[ref24] Crabtree C. Chykina V. (2018). Last name selection in audit studies. Sociol. Sci. 5, 21–28. doi: 10.15195/v5.a2

[ref25] Crabtree C. Gaddis M. S. Guage C. Holbein J. B. Kim J. Y. Marx W. (2022b). Validated names for experimental studies on ethnicity and race. Nat. Sci. Data. 10. 10.1038/s41597-023-01947-0 PMC10006241 36899034

[ref26] Crabtree C. Gaddis S. M. Holbein J. B. Larsen E. N. (2022c). Racially distinctive names signal both race/ethnicity and social class. Sociol. Sci. 9, 454–472. doi: 10.15195/v9.a18

[ref27] Crabtree C. Holbein J. B. Quin Monson J. (2022a). Patient traits shape health-care stakeholders’ choices on how to best allocate life-saving care. Nat. Hum. Behav. 6, 244–257. doi: 10.1038/s41562-021-01280-9, PMID: 35210584

[ref28] Davis S. N. Greenstein T. N. (2009). Gender ideology: Components, predictors, and consequences. Annu. Rev. Sociol. 35, 87–105. doi: 10.1146/annurev-soc-070308-115920

[ref75] Del Rio C. Carlos L. F. Malani P. (2020). Long-term health consequences of COVID-19. JAMA 324, 1723–1724. doi: 10.1001/jama.2020.19719, PMID: 33031513 PMC8019677

[ref003] Doleac J. L. Stein L. C. (2013). The visible hand: Race and online market outcomes. Econ. J. 123, F469–F492.

[ref29] Engelhardt A. M. Utych S. M. (2020). Grand old (Tailgate) party? Partisan discrimination in apolitical settings. Polit. Behav. 42, 769–789. doi: 10.1007/s11109-018-09519-4

[ref30] Enos R. D. (2016). What the demolition of public housing teaches us about the impact of racial threat on political behavior. Am. J. Polit. Sci. 60, 123–142. doi: 10.1111/ajps.12156

[ref31] Filut A. Alvarez M. Carnes M. (2020). Discrimination toward physicians of color: a systematic review. J. Natl. Med. Assoc. 112, 117–140. doi: 10.1016/j.jnma.2020.02.008, PMID: 32197899 PMC7253328

[ref32] Fiorina M. P. Abrams S. J. (2008). Political polarization in the American public. Annu. Rev. Polit. Sci. 11, 563–588. doi: 10.1146/annurev.polisci.11.053106.153836, PMID: 37988103

[ref33] Flage A. (2018). Ethnic and gender discrimination in the rental housing market: Evidence from a meta-analysis of correspondence tests, 2006–2017. J. Hous. Econ. 41, 251–273. doi: 10.1016/j.jhe.2018.07.003

[ref34] Gaddis S. M. (2017). How black are Lakisha and Jamal? Racial perceptions from names used in correspondence audit studies. Sociol. Sci. 4, 469–489. doi: 10.15195/v4.a19

[ref35] Gaddis S. M. Crabtree C. (2021). Correspondence audit studies are necessary to understand discrimination. Available at: SSRN 3813269.

[ref36] Gaddis S. M. Larsen E. N. Crabtree C. Holbein J. H. (2023) Discrimination against Black and Hispanic Americans is highest in hiring and housing contexts: A meta-analysis of correspondence audits. Working paper.

[ref37] Gell-Redman M. Visalvanich N. Crabtree C. Fariss C. J. (2018). It’s all about race: How state legislators respond to immigrant constituents. Polit. Res. Q. 71, 517–531. doi: 10.1177/1065912917749322

[ref38] Giles M. W. Hertz K. (1994). Racial threat and partisan identification. Am. Polit. Sci. Rev. 88, 317–326. doi: 10.2307/2944706

[ref39] Gimpel J. G. Hui I. S. (2015). Seeking politically compatible neighbors? The role of neighborhood partisan composition in residential sorting. Polit. Geogr. 48, 130–142. doi: 10.1016/j.polgeo.2014.11.003

[ref40] Golder S. (2023). The history of anti-Asian discrimination in the United States. Working paper

[ref41] Graham H. (2009). Understanding health inequalities. London, UK: McGraw-Hill education (UK).

[ref42] Guryan J. Charles K. K. (2013). Taste-based or statistical discrimination: the economics of discrimination returns to its roots. Econ. J. 123, F417–F432. doi: 10.1111/ecoj.12080, PMID: 38039795

[ref43] Hainmueller J. Hangartner D. Yamamoto T. (2015). Validating vignette and conjoint survey experiments against real-world behavior. Proc. Natl. Acad. Sci. 112, 2395–2400. doi: 10.1073/pnas.1416587112, PMID: 25646415 PMC4345583

[ref44] Hainmueller J. Hopkins D. J. (2015). The hidden American immigration consensus: A conjoint analysis of attitudes toward immigrants. Am. J. Polit. Sci. 59, 529–548. doi: 10.1111/ajps.12138

[ref45] Haner M. Sloan M. M. Cullen F. T. Graham A. Lero Jonson C. Kulig T. C.. (2020). Making America safe again: Public support for policies to reduce terrorism. Deviant Behav. 42, 1–19. doi: 10.1080/01639625.2020.1738638

[ref46] Hausmann L. R. Hannon M. J. Kresevic D. M. Hanusa B. H. Kwoh C. K. Ibrahim S. A. (2011). Impact of perceived discrimination in health care on patient-provider communication. Med. Care 49:626. doi: 10.1097/MLR.0b013e318215d93c, PMID: 21478769 PMC3117903

[ref47] Healy A. Malhotra N. (2013). Retrospective voting reconsidered. Annu. Rev. Polit. Sci. 16, 285–306. doi: 10.1146/annurev-polisci-032211-212920

[ref48] Hersh E. Ghitza Y. (2018). Mixed partisan households and electoral participation in the United States. PLoS ONE 13:e0203997. doi: 10.1371/journal.pone.0203997, PMID: 30303974 PMC6179382

[ref49] Hibberd C. S. Quan G. M. (2017). Accuracy of preoperative scoring systems for the prognostication and treatment of patients with spinal metastases. Int. Sch. Res. Notices 2017:1320684. doi: 10.1155/2017/1320684 28894788 PMC5574303

[ref50] Higginson I. J. Costantini M. (2002). Accuracy of prognosis estimates by four palliative care teams: a prospective cohort study. BMC Palliat. Care 1, 1–5. doi: 10.1186/1472-684x-1-1 11876829 PMC88964

[ref51] Horiuchi Y. Markovich Z. Yamamoto T. (2022). Does conjoint analysis mitigate social desirability bias?. Polit Anal. 30, 535–549.

[ref52] Huber G. A. Malhotra N. (2017). Political homophily in social relationships: Evidence from online dating behavior. J. Polit. 79, 269–283. doi: 10.1086/687533

[ref53] Hughes D. A. Gell-Redman M. Crabtree C. Krishnaswami N. Rodenberger D. Monge G. (2020). Persistent bias among local election officials. J. Exp. Pol. Sci. 7, 179–187. doi: 10.1017/XPS.2019.23

[ref54] Iyengar S. Lelkes Y. Levendusky M. Malhotra N. Westwood S. J. (2019). The origins and consequences of affective polarization in the United States. Annu. Rev. Polit. Sci. 22, 129–146. doi: 10.1146/annurev-polisci-051117-073034

[ref55] Jenke L. Bansak K. Hainmueller J. Hangartner D. (2021). Using eye-tracking to understand decision-making in conjoint experiments. Polit. Anal. 29, 75–101. doi: 10.1017/pan.2020.11

[ref56] Kalkan K. O. Layman G. C. Uslaner E. M. (2009). “Bands of others”? Attitudes toward Muslims in contemporary American society. J. Polit. 71, 847–862. doi: 10.1017/S0022381609090756

[ref57] Kam C. D. Deichert M. (2020). Boycotting, buycotting, and the psychology of political consumerism. J. Polit. 82, 72–88. doi: 10.1086/705922

[ref58] Kawachi I. Subramanian S. V. Almeida-Filho N. (2002). A glossary for health inequalities. J. Epidemiol. Community Health 56, 647–652. doi: 10.1136/jech.56.9.647, PMID: 12177079 PMC1732240

[ref61] Kouta C. Kaite C. P. (2011). Gender discrimination and nursing: α literature review. J. Prof. Nurs. 27, 59–63. doi: 10.1016/j.profnurs.2010.10.006, PMID: 21272837

[ref62] Lajevardi N. (2020). Outsiders at home: The politics of American Islamophobia. Cambridge, MA: Cambridge University Press.

[ref63] Lauderdale D. S. Wen M. Jacobs E. A. Kandula N. R. (2006). Immigrant perceptions of discrimination in health care: the California Health Interview Survey 2003. Med. Care 44, 914–920. doi: 10.1097/01.mlr.0000220829.87073.f7, PMID: 17001262

[ref64] Leung K. Cheng K. Zhang J. Cheng Y. Nguyen Cao V. H. Ioku S.. (2021). How Asians react to discrimination does not depend on their party identification. Socius 7:23780231211048023. doi: 10.1177/237802312110480

[ref65] Lippens L. Vermeiren S. Baert S. (2023). The state of hiring discrimination: A meta-analysis of (almost) all recent correspondence experiments. Eur. Econ. Rev. 151:104315. doi: 10.1016/j.euroecorev.2022.104315

[ref0004] Lippens L. Baert S. Ghekiere A. Verhaeghe P. P. Derous E. (2022). Is labour market discrimination against ethnic minorities better explained by taste or statistics? A systematic review of the empirical evidence. J. Ethn. Migr. Stud. 48, 4243–4276.

[ref66] Morgan K. J. Campbell A. L. (2011). Delegated Governance in the Affordable Care Act. J. Health Polit. Policy Law 36, 387–391. doi: 10.1215/03616878-1271000, PMID: 21673235

[ref67] Mummolo J. Nall C. (2017). Why partisans do not sort: The constraints on political segregation. J. Polit. 79, 45–59. doi: 10.1086/687569, PMID: 37988071

[ref68] Neilson L. A. (2010). Boycott or buycott? Understanding political consumerism. J. Consum. Behav. 9, 214–227. doi: 10.1002/cb.313, PMID: 38050708

[ref69] Nicholson S. P. Coe C. M. Emory J. Song A. V. (2016). The politics of beauty: The effects of partisan bias on physical attractiveness. Polit. Behav. 38, 883–898. doi: 10.1007/s11109-016-9339-7

[ref70] Pager D. Shepherd H. (2008). The sociology of discrimination: Racial discrimination in employment, housing, credit, and consumer markets. Annu. Rev. Sociol. 34, 181–209. doi: 10.1146/annurev.soc.33.040406.131740, PMID: 20689680 PMC2915460

[ref71] Paxton P. Kunovich S. Hughes M. M. (2007). Gender in politics. Annu. Rev. Sociol. 33, 263–284. doi: 10.1146/annurev.soc.33.040406.131651, PMID: 38077986

[ref72] Pololi L. Cooper L. Carr P. Cooper L. A. (2010). Race, disadvantage and faculty experiences in academic medicine. J. Gen. Intern. Med. 25, 1363–1369. doi: 10.1007/s11606-010-1478-7, PMID: 20697960 PMC2988158

[ref006] Porter E. Velez Y. R. (2022). Placebo selection in survey experiments: An agnostic approach. Polit Anal. 30, 481–494.

[ref73] Quillian L. Lee J. J. (2023). Trends in racial and ethnic discrimination in hiring in six Western countries. Proc. Natl. Acad. Sci. 120:e2212875120. doi: 10.1073/pnas.2212875120, PMID: 36719918 PMC9963383

[ref74] Quillian L. Pager D. Hexel O. Midtbøen A. H. (2017). Meta-analysis of field experiments shows no change in racial discrimination in hiring over time. Proc. Natl. Acad. Sci. 114, 10870–10875. doi: 10.1073/pnas.1706255114, PMID: 28900012 PMC5642692

[ref77] Sakamoto A. Takei I. Woo H. (2012). The myth of the model minority myth. Sociol. Spectr. 32, 309–321. doi: 10.1080/02732173.2012.664042, PMID: 37883043

[ref78] Salisbury C. J. (1989). How do people choose their doctor? Br. Med. J. 299, 608–610. 2508824 10.1136/bmj.299.6699.608 PMC1837422

[ref79] Santos R. Gravelle H. Propper C. (2017). Does quality affect patients’ choice of doctor? Evidence from England. Econ. J. 127, 445–494. doi: 10.1111/ecoj.12282 PMC5349292 28356602

[ref80] Schwab S. (1986). Is statistical discrimination efficient? Am. Econ. Rev. 76, 228–234.

[ref81] Shafranek R. M. (2021). Political considerations in nonpolitical decisions: a conjoint analysis of roommate choice. Polit. Behav. 43, 271–300. doi: 10.1007/s11109-019-09554-9

[ref82] Weaver V. M. Prowse G. (2020). Racial authoritarianism in US democracy. Science 369, 1176–1178. doi: 10.1126/science.abd7669, PMID: 32883857

[ref005] Williams D. R. Lawrence J. A. Davis B. A. Vu C. (2019). Understanding how discrimination can affect health. Health Serv. Res. 54, 1374–1388. 31663121 10.1111/1475-6773.13222 PMC6864381

[ref002] Yinger J. (1995). Closed doors, opportunities lost: The continuing costs of housing discrimination. New York, NY: Russell Sage Foundation.

[ref83] Zschirnt E. (2016). Measuring hiring discrimination–a history of field experiments in discrimination research. NCCR-on the move, Working Paper Series 7

