# Supplementary material for: Americans do not select their doctors based on race
Source: Front Sociol. 2024 Jan 24;8:1191080. doi: 10.3389/fsoc.2023.1191080 (PMC10847235; doi:10.3389/fsoc.2023.1191080)
Supplement: Supplementary file 1 [file Table_1.docx]

**Supplementary Information for “Americans do not discriminate against doctors based on their race”**

**SI 1: Study AMCEs**

In this SI, we report the average marginal component effects from our study. They are substantively similar to the marginal means that we report in the main text.

**Figure SI 1a: Public Preferences for Doctors**


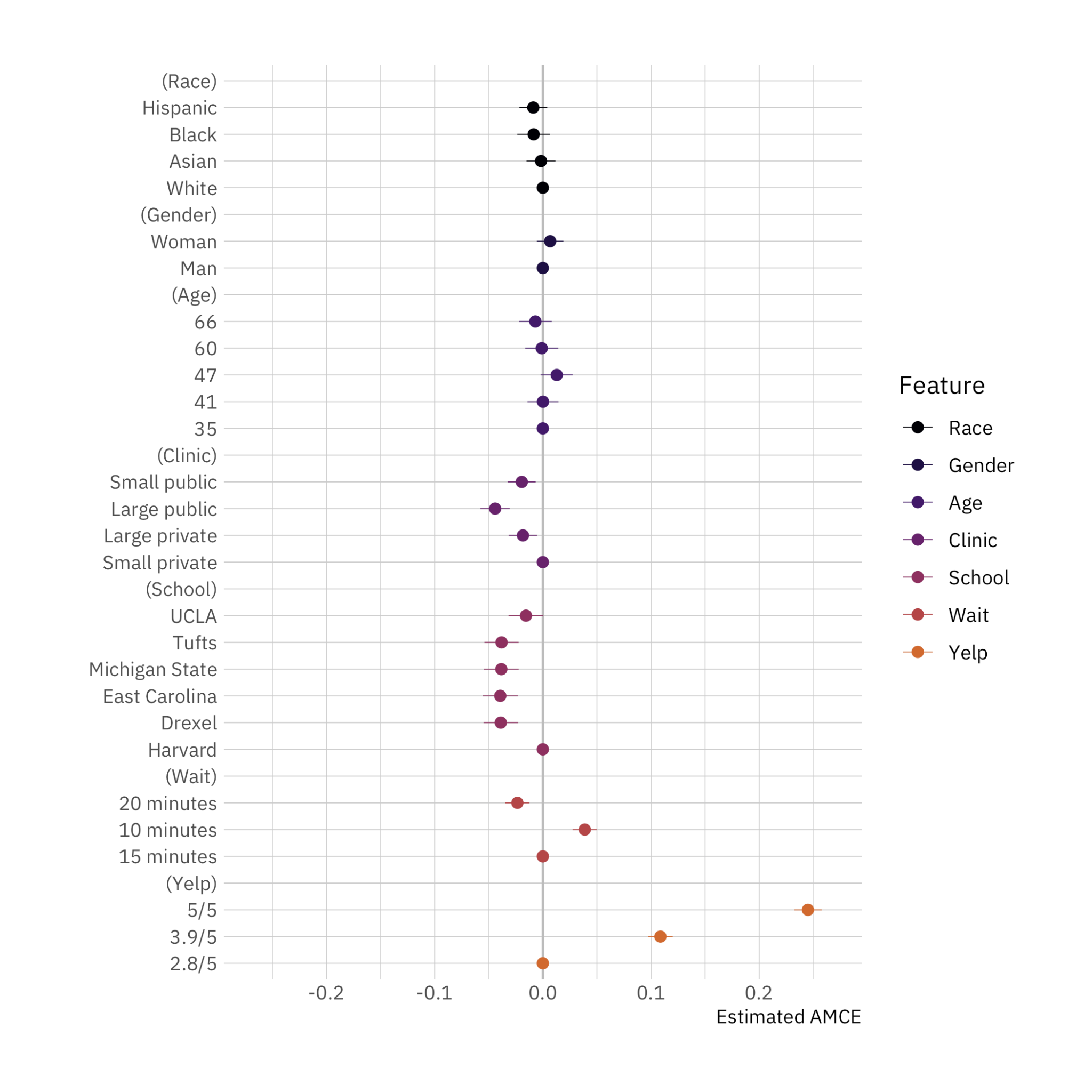


*Note:* AMCE plot for the effect of doctor attributes on survey respondent selection. The circles represent the marginal means while the thin bars denote 95% confidence intervals. Coefficients on the left side of the grey line at 50% indicate that respondents are, all-else-equal, less likely to choose a doctor with the given characteristics on the vertical axis; those on the right are, all-else-equal, more likely to choose a doctor with the given characteristic. The unit of analysis is the respondent-choice profile. Hence, the $N$ reported in our models below is the number of respondents (1,498) multiplied by the number of pairwise choices (15) and individuals within those pairs (2). N = 44,940. Confidence intervals are calculated based on standard errors clustered by respondent (Bansak 2021).

**SI 2: Study treatment effect heterogeneity**

In this SI, we report the results from several additional treatment effect heterogeneity tests. As described in the main text, we explore possible treatment effect heterogeneity by looking at group differences across respondent education, gender, and views on racial discrimination. We find no compelling, consistent differences across groups, especially after adjusting p-values for multiple comparisons. Ultimately, while there is some small variation in our treatment effect heterogeneity results, the overall trend is clear: members of the public do not provide differential treatment to doctors in our task based on race.

**Figure SI 2a: Public Preferences for Doctors by Education**


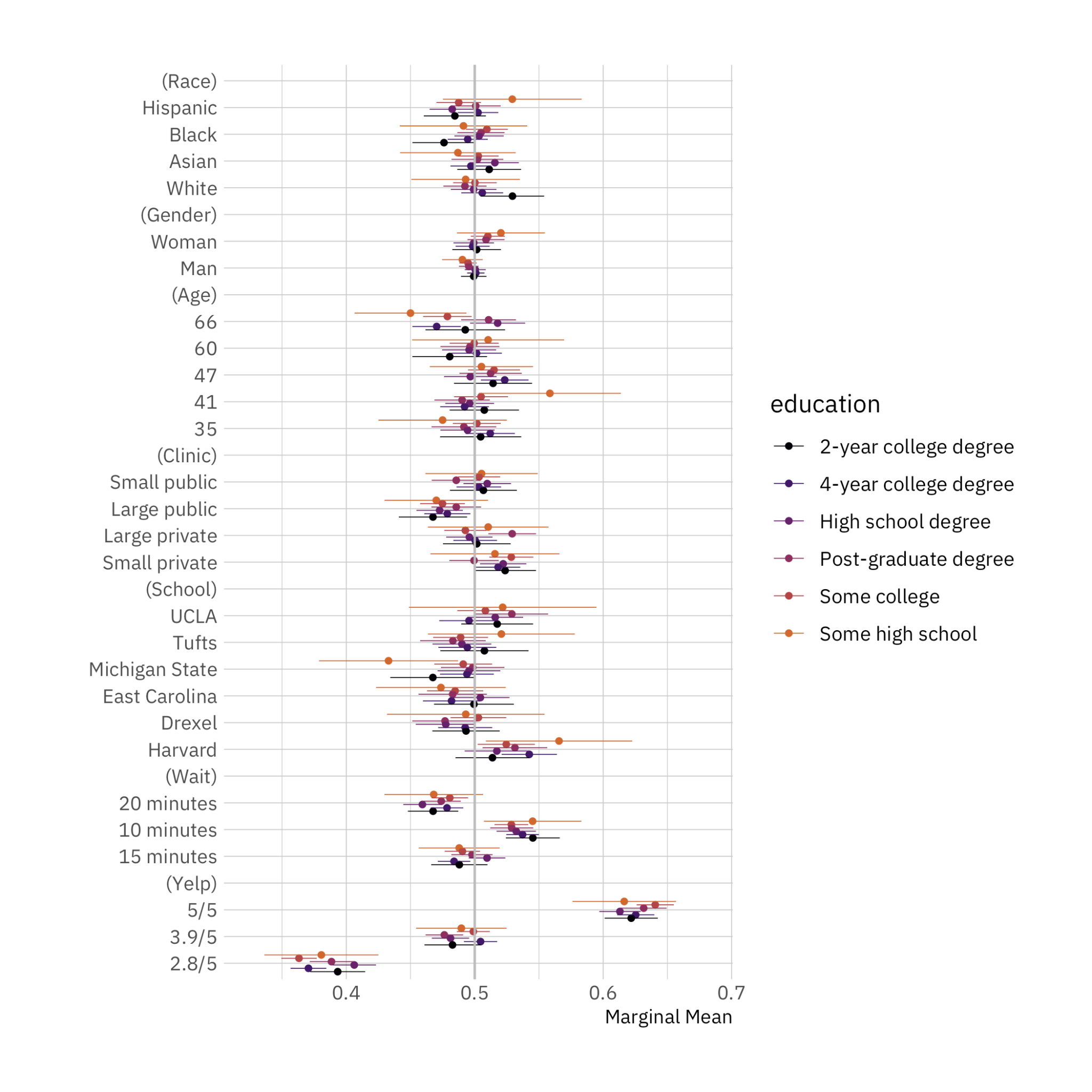


*Note:* Conditional marginal means for the effect of doctor attributes on survey respondent selection by respondent education level. The circles represent the marginal means while the thin bars denote 95% confidence intervals. Coefficients on the left side of the grey line at 50% indicate that respondents are, all-else-equal, less likely to choose a doctor with the given characteristics on the vertical axis; those on the right are, all-else-equal, more likely to choose a doctor with the given characteristic. The unit of analysis is the respondent-choice profile. The N reported in our models below is the number of respondents (1,498) multiplied by the number of pairwise choices (15) and individuals within those pairs (2). N = 44,940. Confidence intervals are calculated based on standard errors clustered by respondent (Bansak 2021).

**Figure SI 2b: Public Preferences for Doctors by Gender**


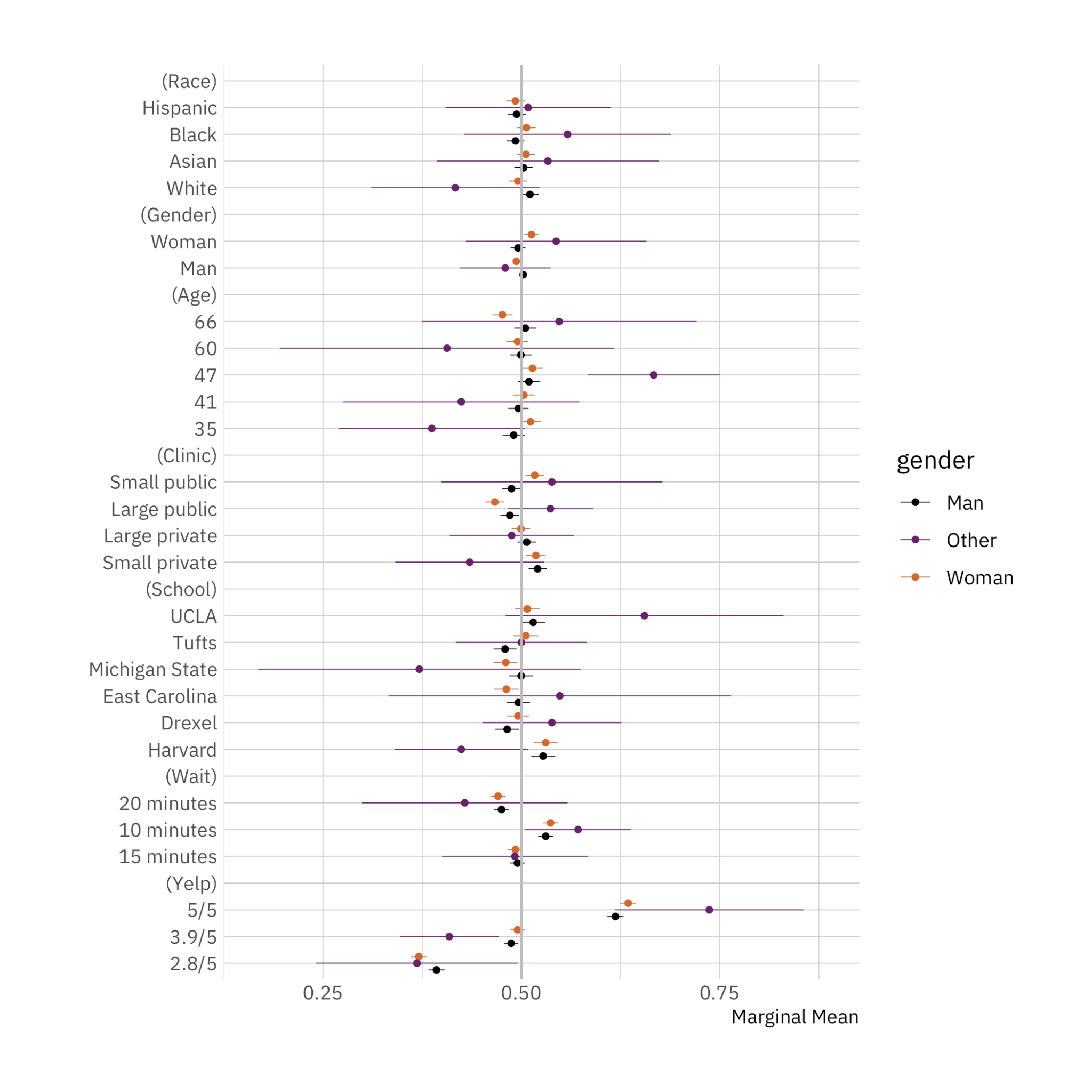


*Note:* Conditional marginal means for the effect of doctor attributes on survey respondent selection by respondent gender. The circles represent the marginal means while the thin bars denote 95% confidence intervals. Coefficients on the left side of the grey line at 50% indicate that respondents are, all-else-equal, less likely to choose a doctor with the given characteristics on the vertical axis; those on the right are, all-else-equal, more likely to choose a doctor with the given characteristic. The unit of analysis is the respondent-choice profile. The N reported in our models below is the number of respondents (1,498) multiplied by the number of pairwise choices (15) and individuals within those pairs (2). N = 44,940. Confidence intervals are calculated based on standard errors clustered by respondent (Bansak 2021).

**Figure SI 2c: Public Preferences for Doctors by Views on Racism**


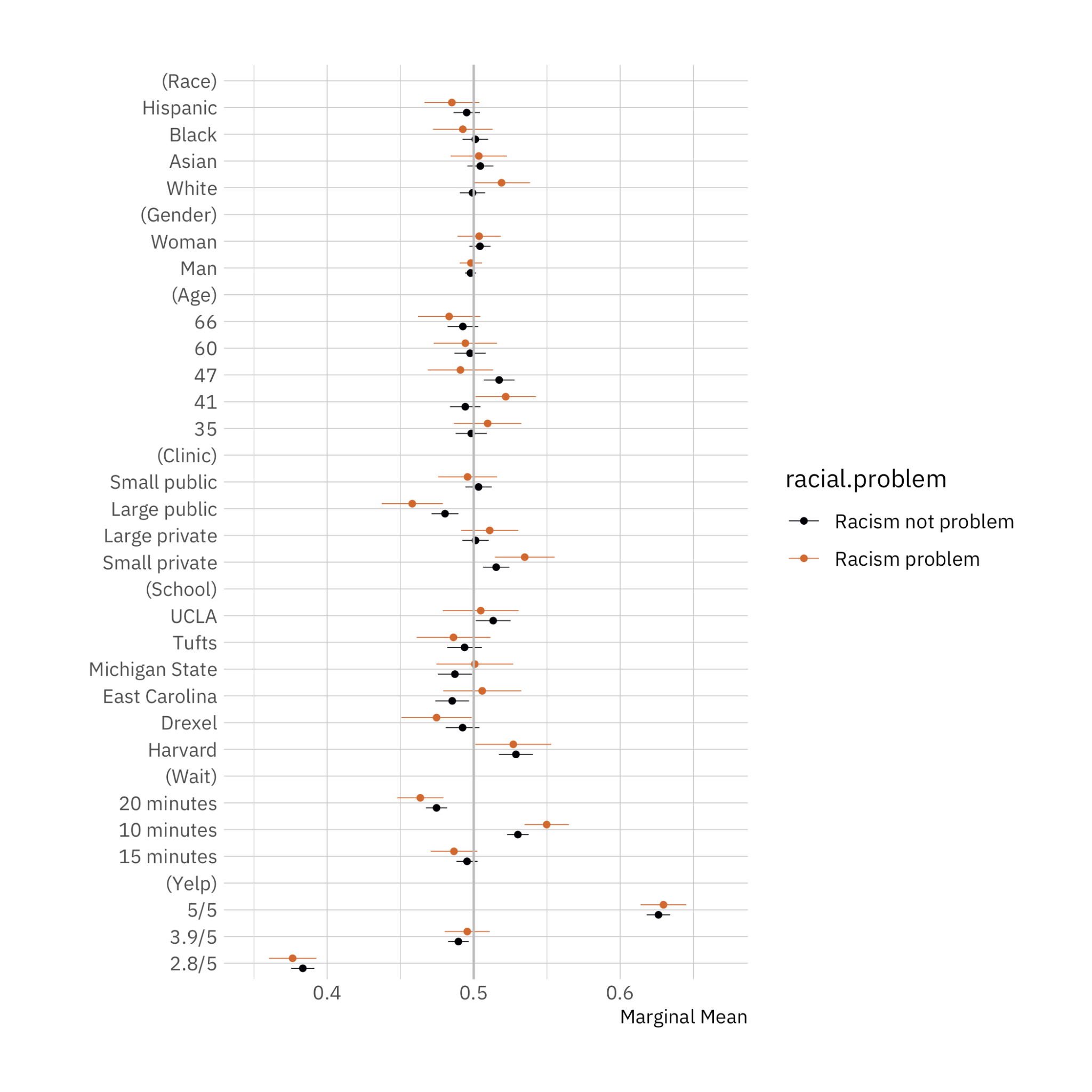


*Note:* Conditional marginal means for the effect of doctor attributes on survey respondent selection by respondent views on racism. The circles represent the marginal means while the thin bars denote 95% confidence intervals. Coefficients on the left side of the grey line at 50% indicate that respondents are, all-else-equal, less likely to choose a doctor with the given characteristics on the vertical axis; those on the right are, all-else-equal, more likely to choose a doctor with the given characteristic. The unit of analysis is the respondent-choice profile. The N reported in our models below is the number of respondents (1,498) multiplied by the number of pairwise choices (15) and individuals within those pairs (2). N = 44,940. Confidence intervals are calculated based on standard errors clustered by respondent (Bansak 2021).

It appears as if, in general, individuals who say that racism is a problem are more likely to select white doctors. We don’t have strong theoretical expectations as to why this might be the case, but it could be that this question item is one where respondents are highly likely to engage in preference falsification, and that those individuals who are most likely to do this are also the most likely to exhibit implicit biases against doctors from historically marginalized groups. We think that future work should try to examine this possibility.

**SI 3: Study demographics**

**
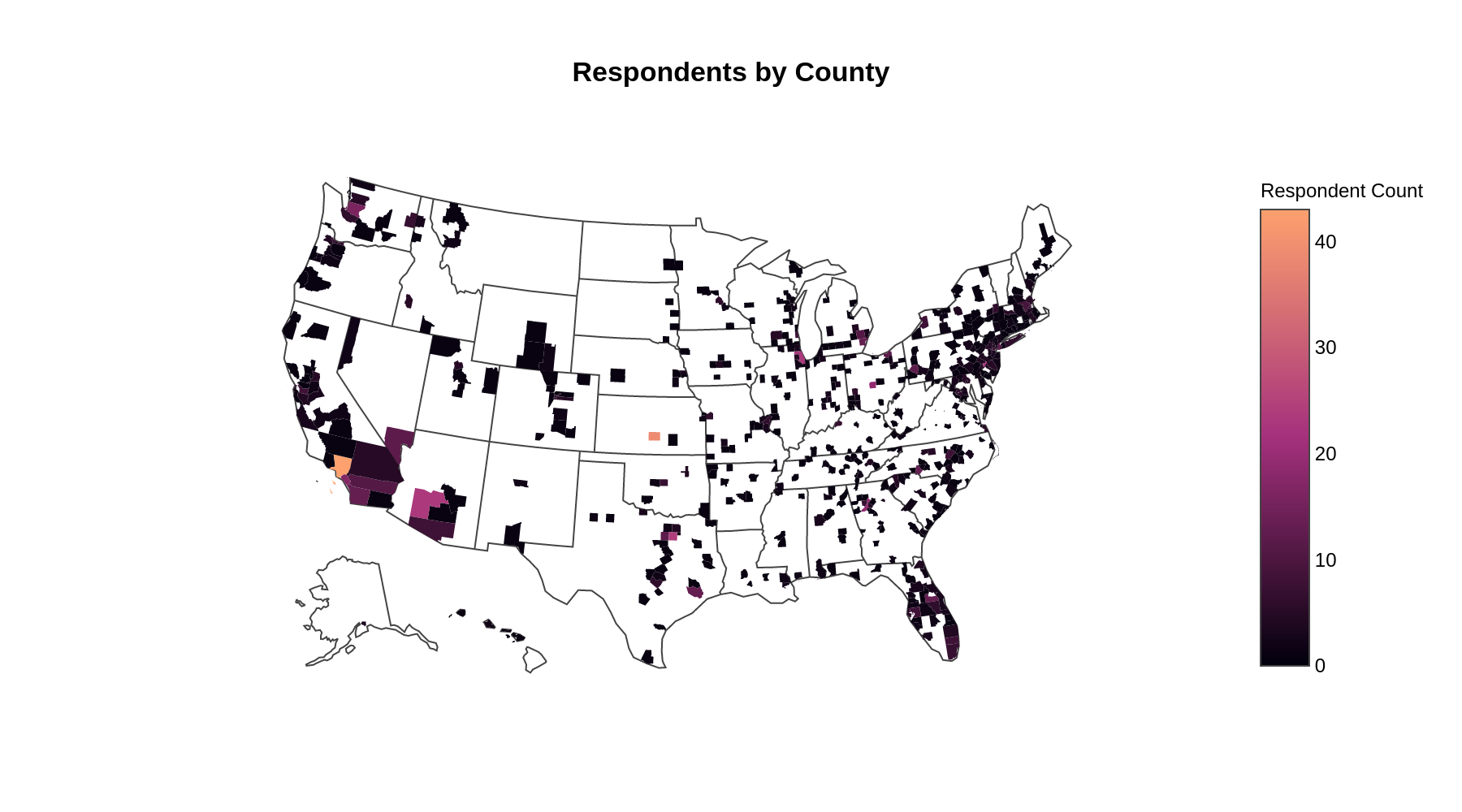
**

**
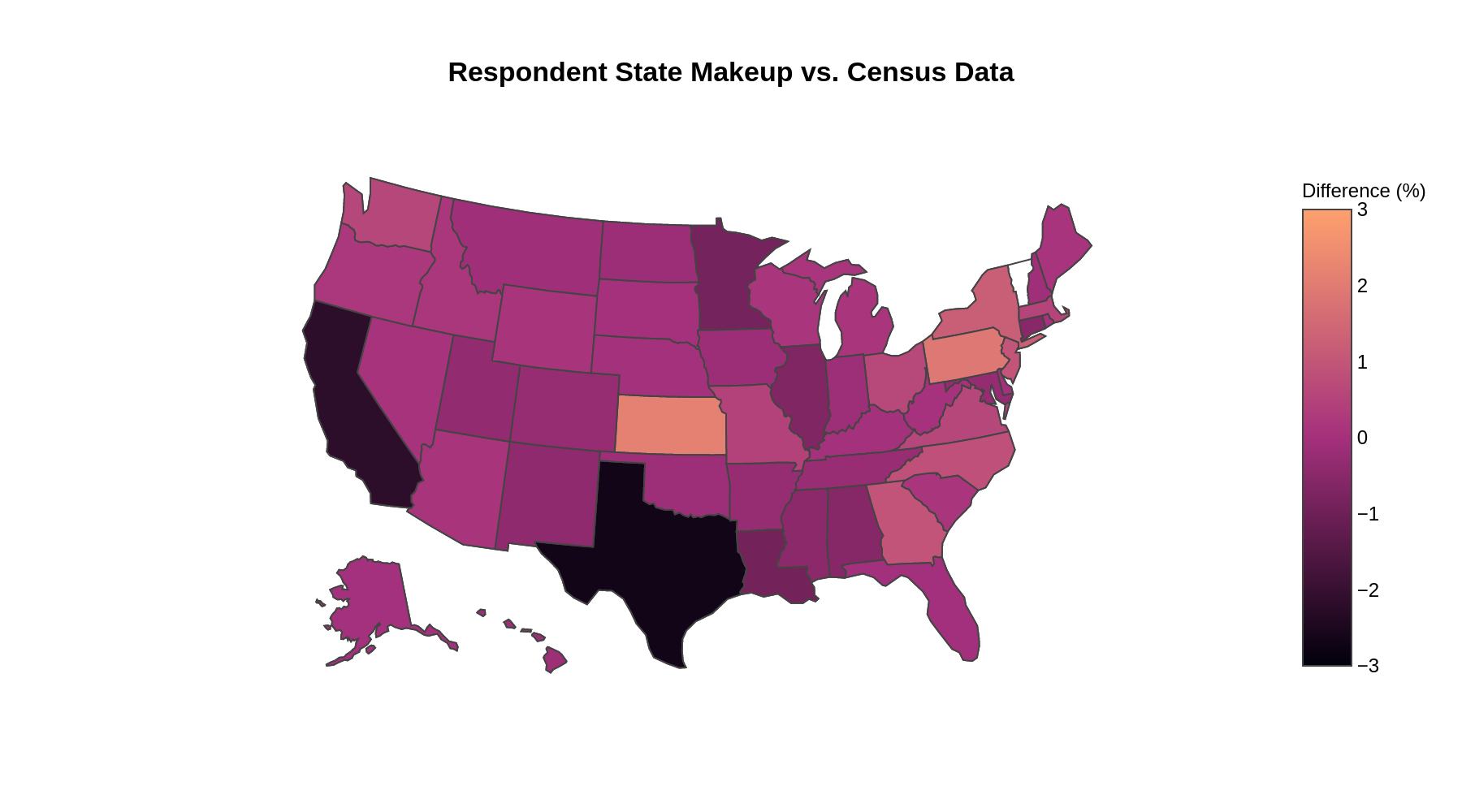
**

**
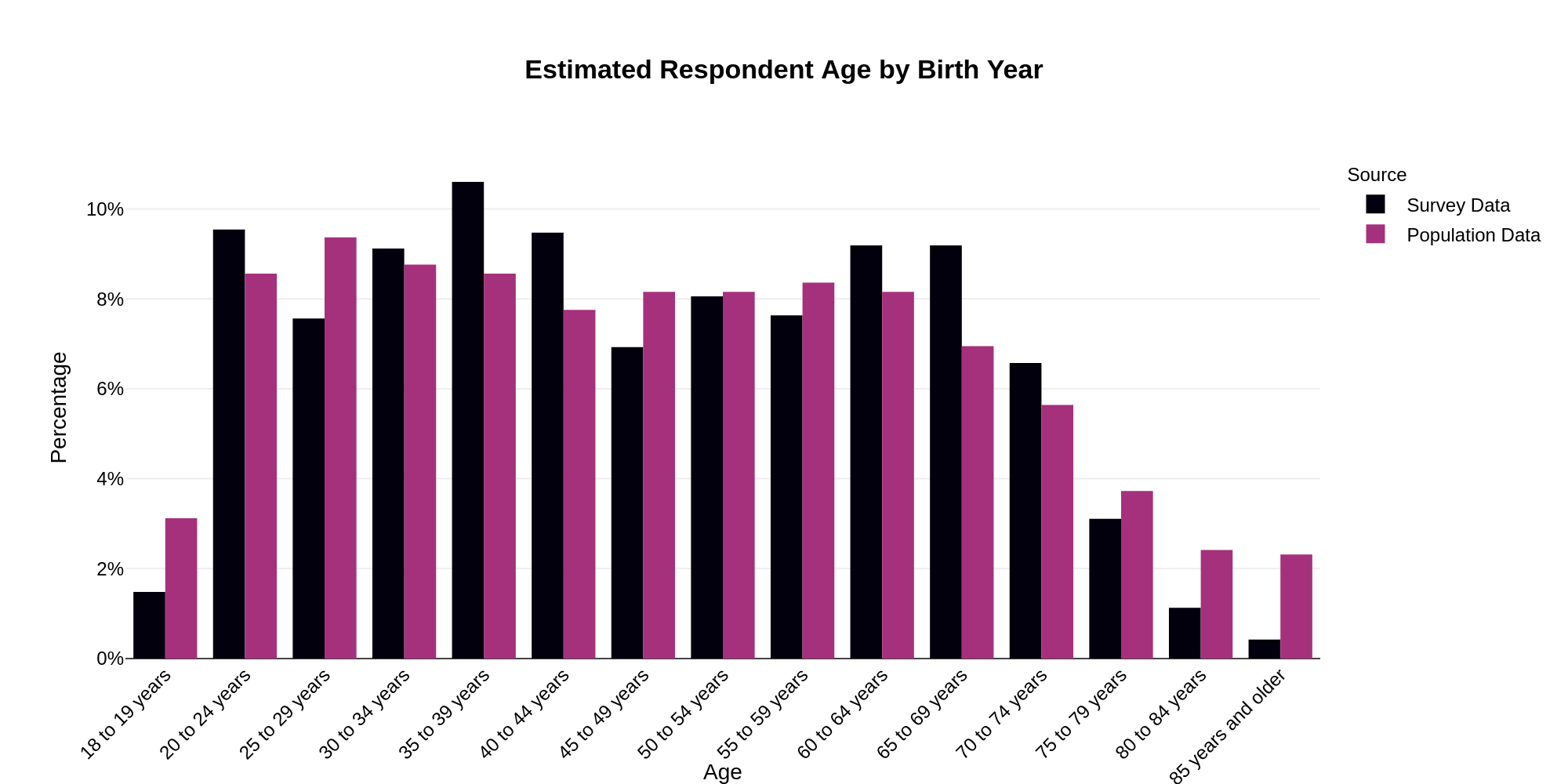
**

**
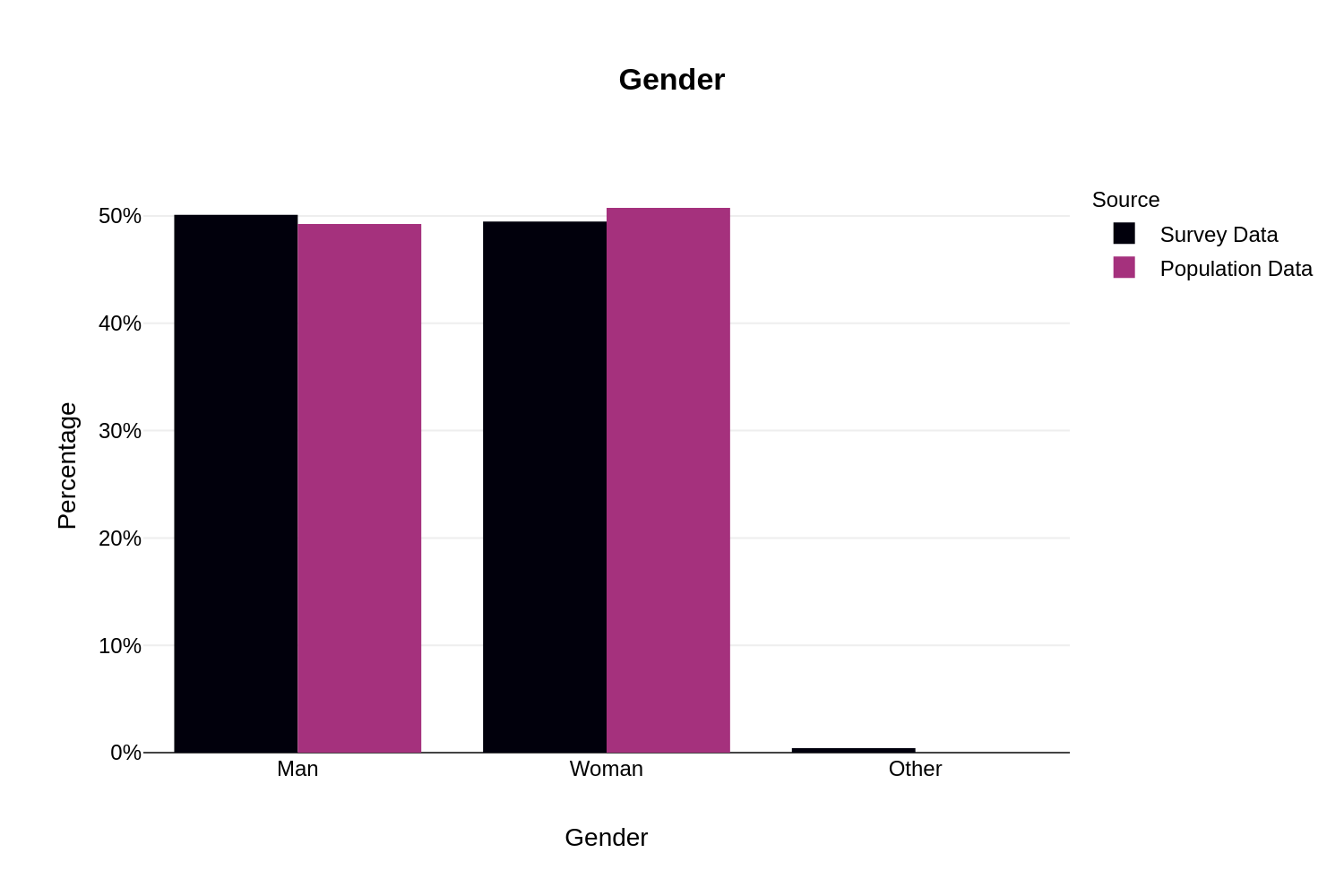
**

**
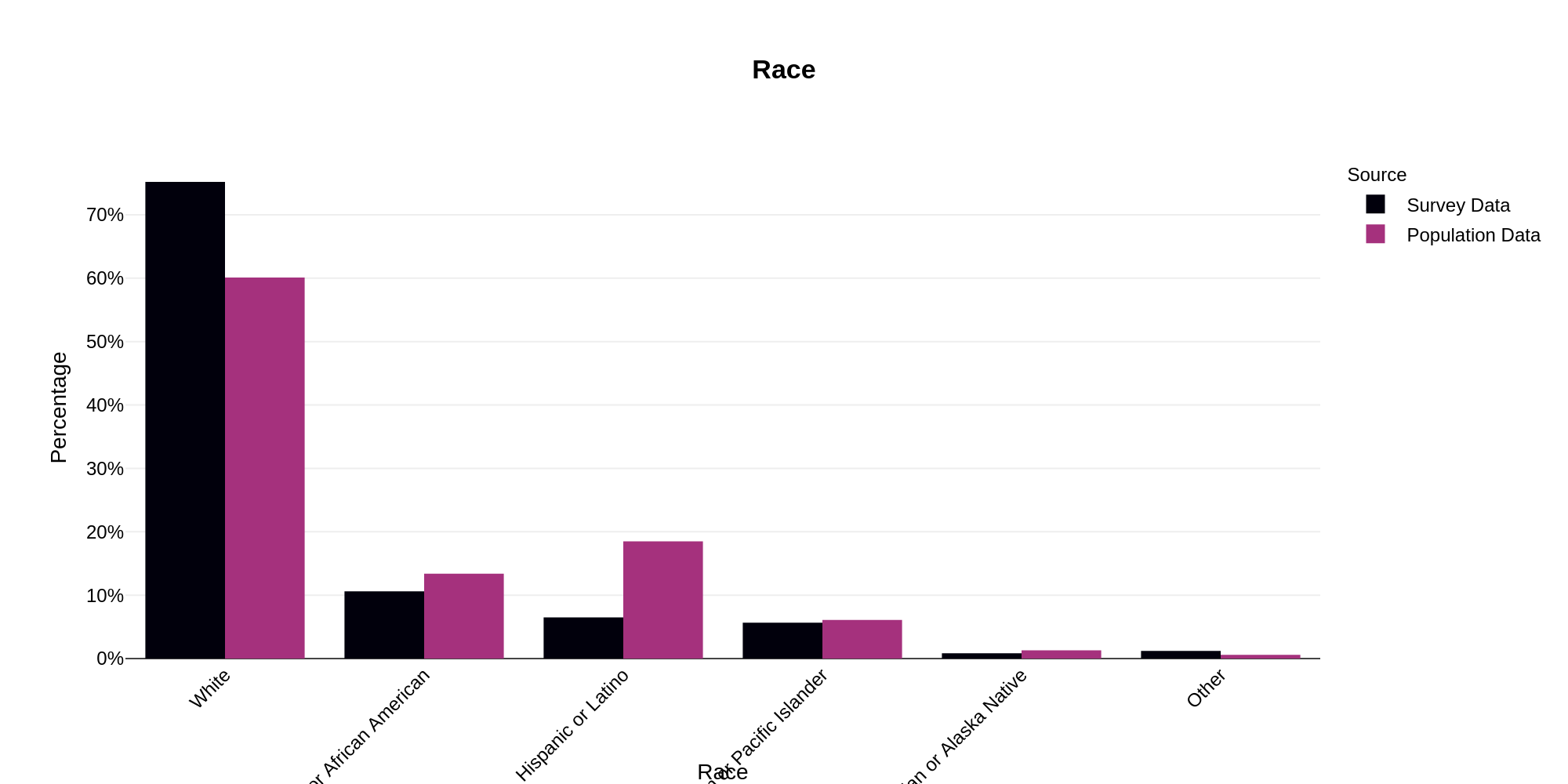
**

**
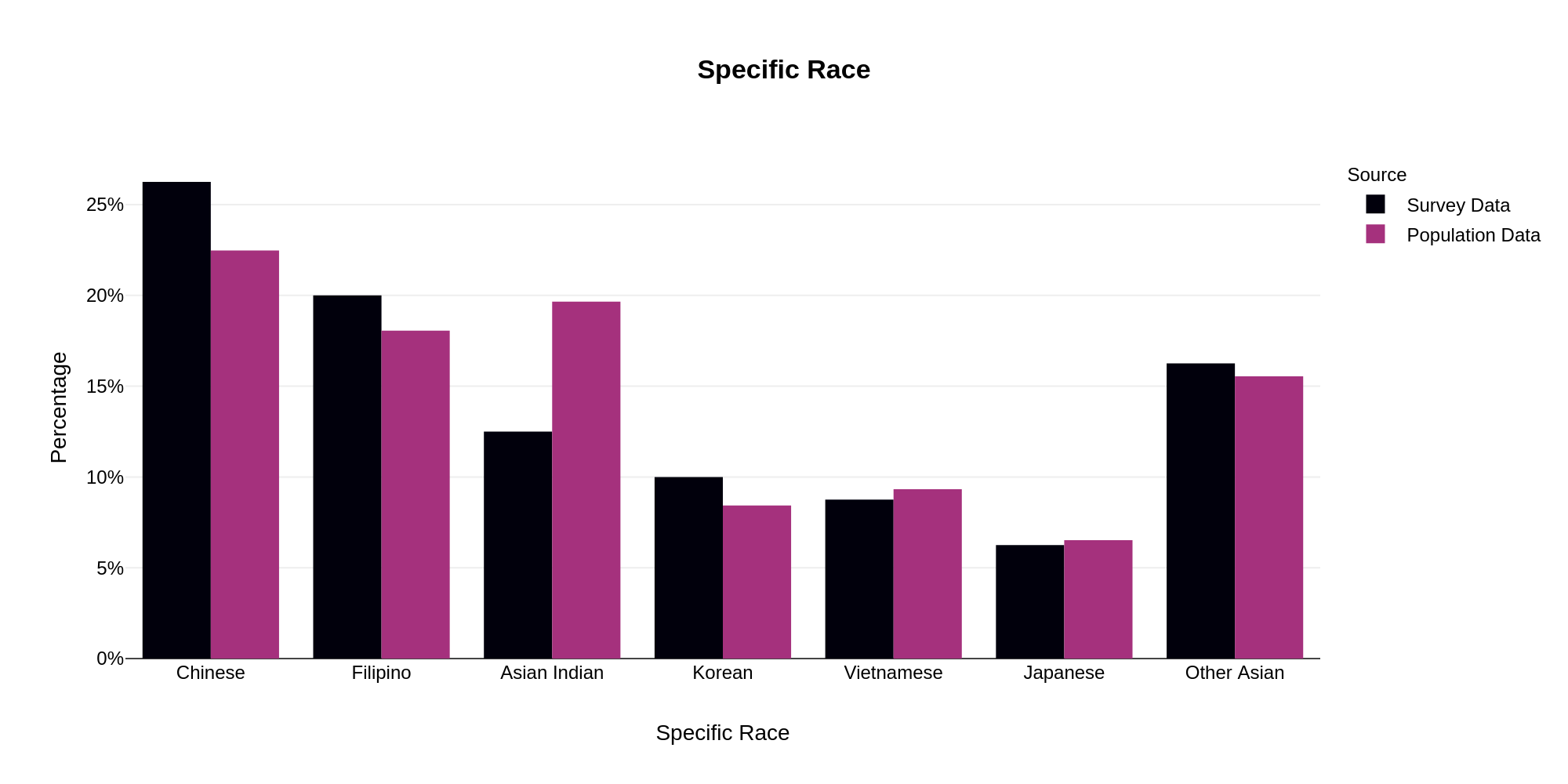
**

**
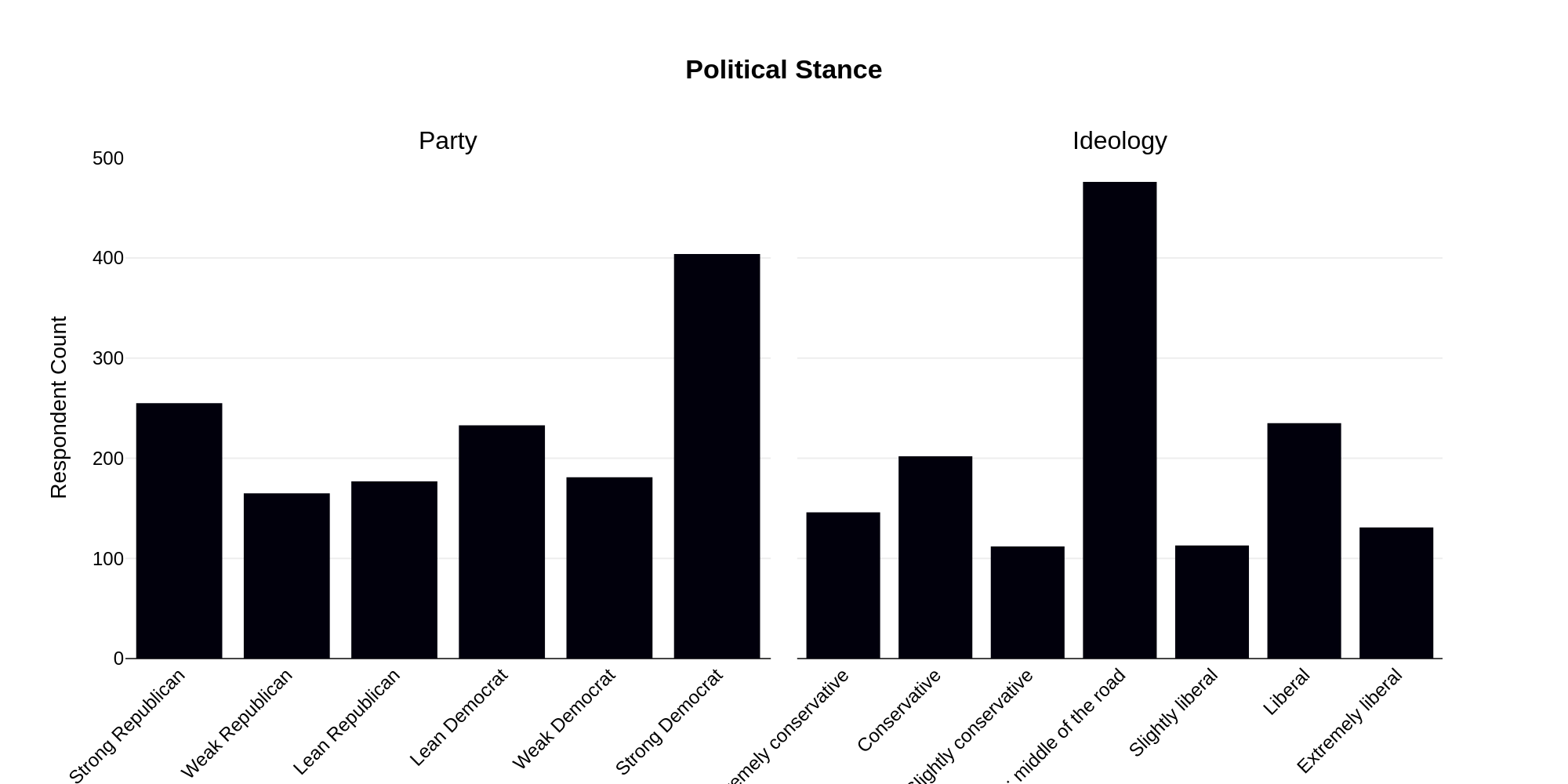
**

**
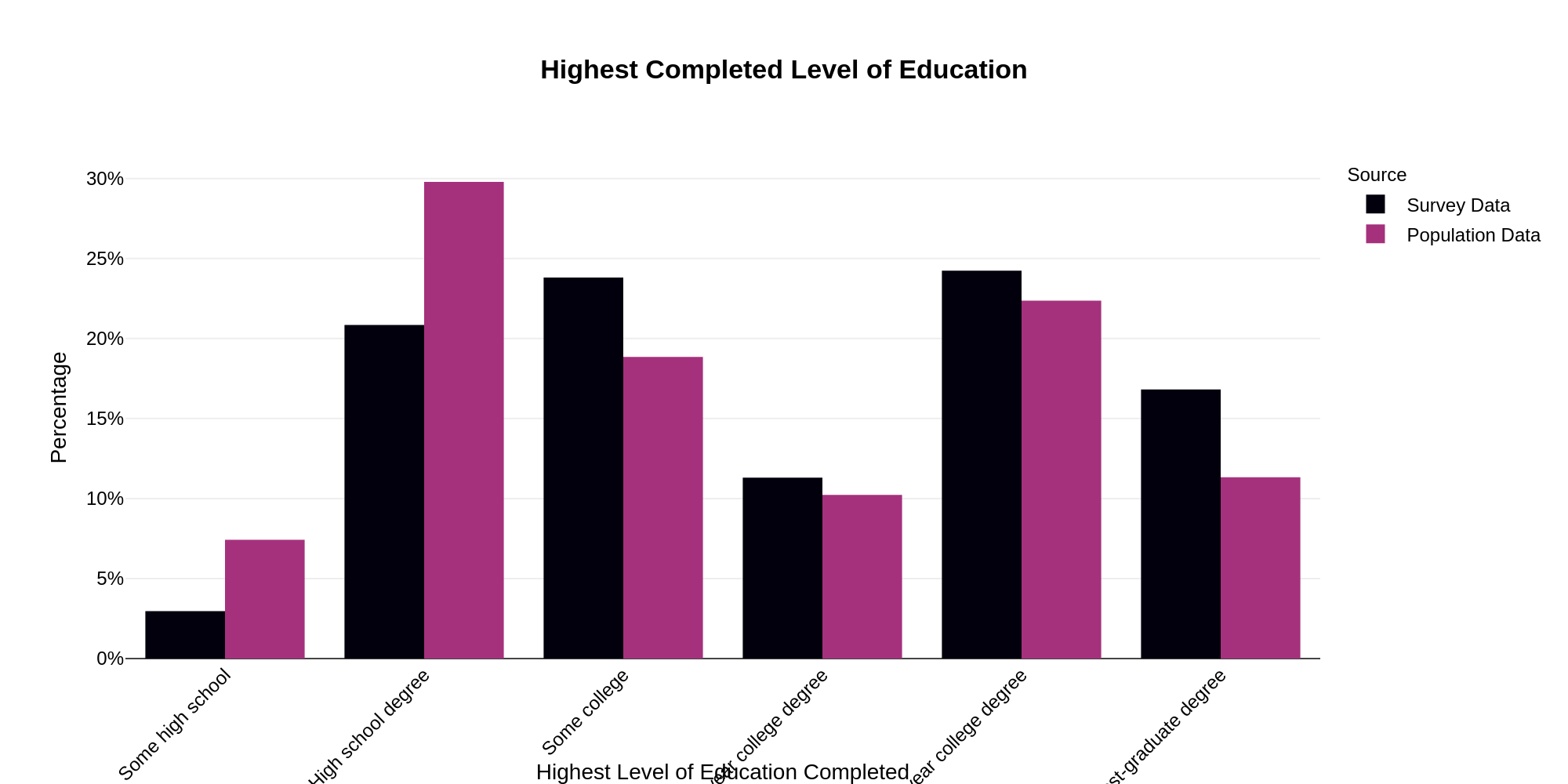
**

**
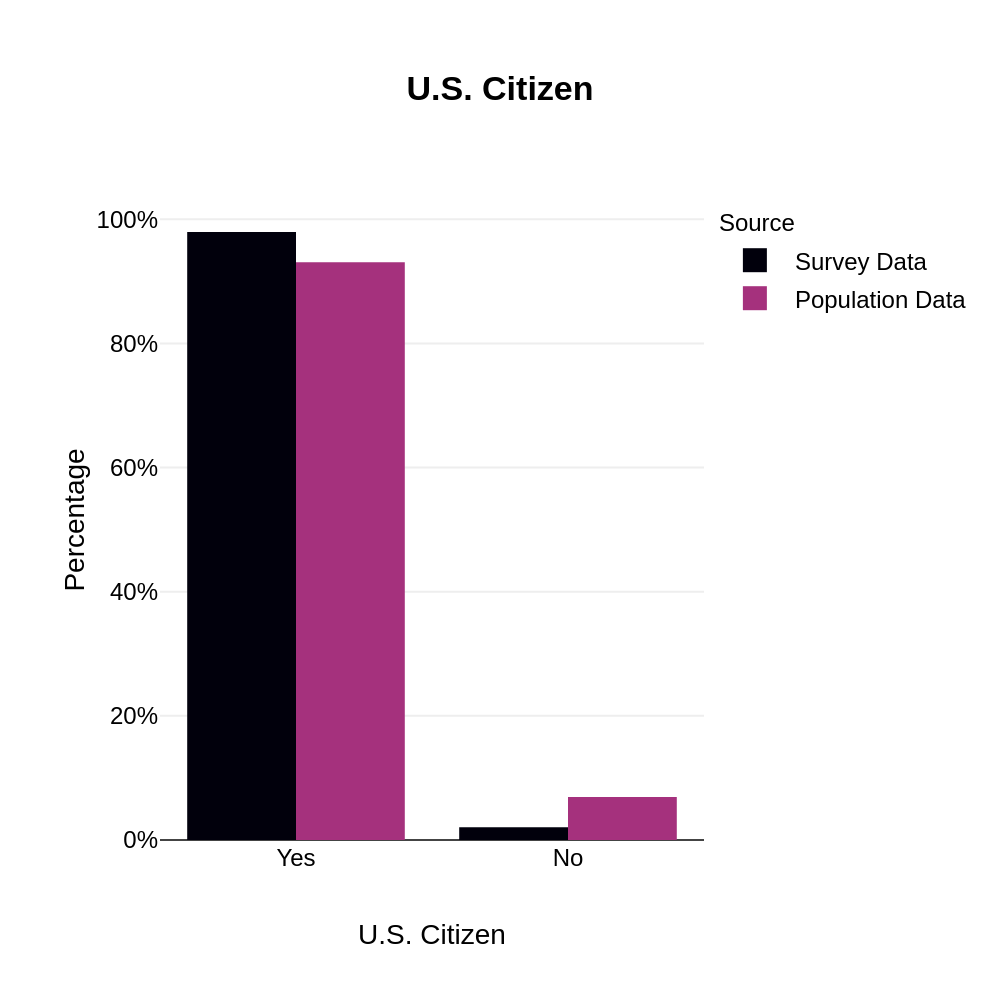
**

**
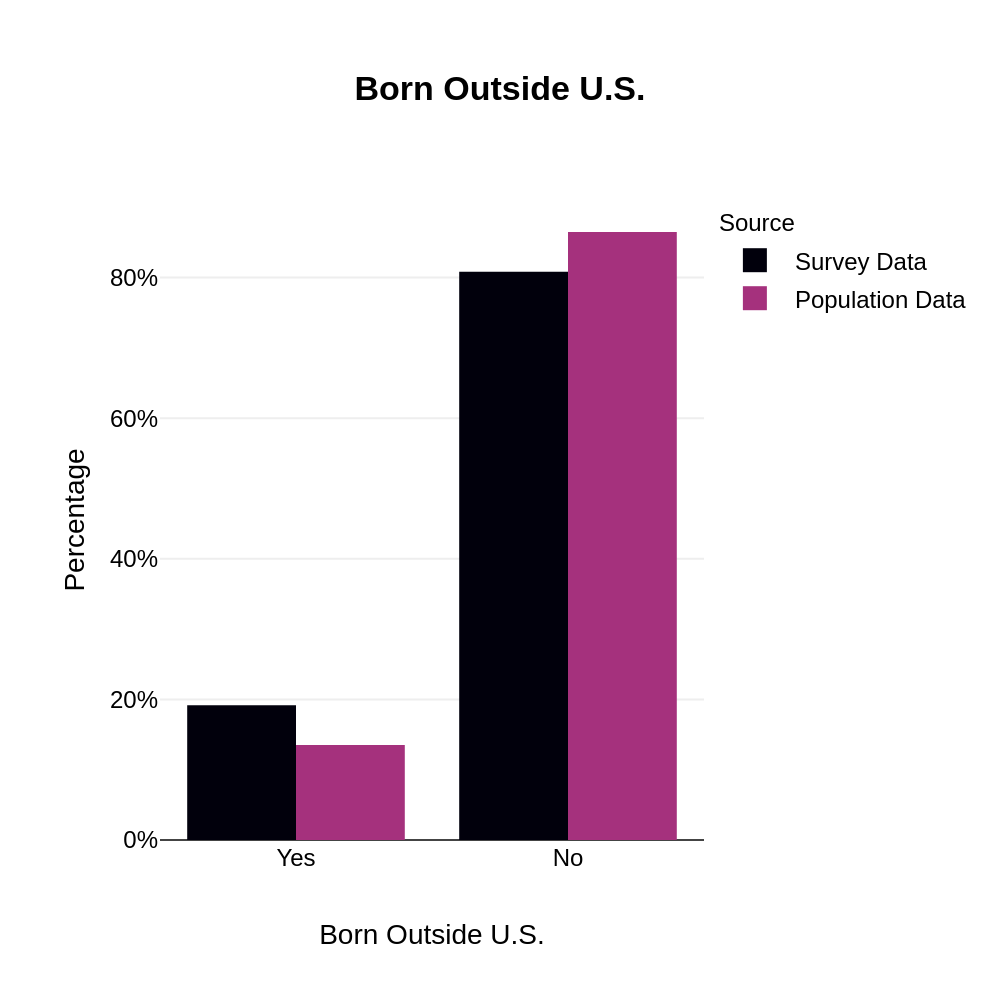
**
